# The Number of Patients and Events Required to Limit the Risk of Overestimation of Intervention Effects in Meta-Analysis—A Simulation Study

**DOI:** 10.1371/journal.pone.0025491

**Published:** 2011-10-18

**Authors:** Kristian Thorlund, Georgina Imberger, Michael Walsh, Rong Chu, Christian Gluud, Jørn Wetterslev, Gordon Guyatt, Philip J. Devereaux, Lehana Thabane

**Affiliations:** 1 Department of Clinical Epidemiology and Biostatistics, McMaster University, Ontario, Canada; 2 Copenhagen Trial Unit, Centre for Clinical Intervention Research, Department 3344, Rigshospitalet, Copenhagen University Hospital, Copenhagen, Denmark; 3 Biostatistics Unit, Father Sean O'Sullivan Research Centre, St Joseph's Healthcare Hamilton, Hamilton, Canada; 4 Public Health Research Institute, McMaster University, Hamilton, Canada; University of Modena and Reggio Emilia, Italy

## Abstract

**Background:**

Meta-analyses including a limited number of patients and events are prone to yield overestimated intervention effect estimates. While many assume bias is the cause of overestimation, theoretical considerations suggest that random error may be an equal or more frequent cause. The independent impact of random error on meta-analyzed intervention effects has not previously been explored. It has been suggested that surpassing the optimal information size (i.e., the required meta-analysis sample size) provides sufficient protection against overestimation due to random error, but this claim has not yet been validated.

**Methods:**

We simulated a comprehensive array of meta-analysis scenarios where no intervention effect existed (i.e., relative risk reduction (RRR) = 0%) or where a small but possibly unimportant effect existed (RRR = 10%). We constructed different scenarios by varying the control group risk, the degree of heterogeneity, and the distribution of trial sample sizes. For each scenario, we calculated the probability of observing overestimates of RRR>20% and RRR>30% for each cumulative 500 patients and 50 events. We calculated the cumulative number of patients and events required to reduce the probability of overestimation of intervention effect to 10%, 5%, and 1%. We calculated the optimal information size for each of the simulated scenarios and explored whether meta-analyses that surpassed their optimal information size had sufficient protection against overestimation of intervention effects due to random error.

**Results:**

The risk of overestimation of intervention effects was usually high when the number of patients and events was small and this risk decreased exponentially over time as the number of patients and events increased. The number of patients and events required to limit the risk of overestimation depended considerably on the underlying simulation settings. Surpassing the optimal information size generally provided sufficient protection against overestimation.

**Conclusions:**

Random errors are a frequent cause of overestimation of intervention effects in meta-analyses. Surpassing the optimal information size will provide sufficient protection against overestimation.

## Introduction

Systematic reviews and meta-analyses combining evidence from several high-quality randomized clinical trials (RCTs) are generally considered the highest level of evidence for effects of interventions [1]–[3]. Many systematic reviews address questions important and pressing to a large group of patients and clinicians. Therefore, these analyses are often conducted at a stage when the evidence on a particular question is still limited. Such meta-analyses lack the precision (i.e., are underpowered) to establish realistic intervention effects with a high level of confidence [4]–[11]. Yet, it is not infrequently that such preliminary meta-analyses yield apparently large intervention effect estimates which, if meeting the conventional criterion for statistical significance (i.e., p≤0.05), can appear compelling [4]–[11]. Empirical studies suggest that when more evidence is accumulated over time, many of these ‘early’ large apparent intervention effects turn out to be substantial overestimates [4]–[6], [12]. Meta-analysis authors often assume that time-lag, publication bias, methodological bias, or outcome reporting bias are the main cause(s) of early overestimation, but theoretical considerations suggest that lack of precision may be an equally or more frequent cause [3]–[11], [13].

As authors and users of meta-analyses and systematic reviews, we wish to avoid the mistake of trusting spuriously large meta-analyzed intervention effects. Because precision (and power) is highly correlated with the cumulative number of patients and events, some authors have recommended that meta-analyzed intervention effect estimates should be interpreted in relation to the cumulative number of patients or events [6]–[9], [14]–[17]. In particular, a *required* or an *optimal* information size (OIS, analogous to a required sample size in a clinical trial) has been proposed for meta-analysis [9], [15]–[17]. While we find this proposal highly useful, the optimal information size does not provide insight into the degree and likelihood of overestimation of intervention effects that one can expect at various preceding stages of a meta-analysis. Further, it is unknown whether conventional information size requirements (i.e., α = 5%, β = 10%, and plausible a priori assumptions about the intervention effect, control group risk, and degree of heterogeneity), provide sufficient protection against overestimation of meta-analyzed intervention effects caused by random errors (imprecision). The existing empirical studies on this topic are, unfortunately, limited by their respective sample sizes (the number of meta-analyses studied empirically), and thus, do not provide a reliable basis for assessing the expected degree and likelihood of overestimation at various stages evidence accumulation. Further, because the impact of bias (systematic error) is next to impossible to infer with certainty in individual meta-analyses, it is also difficult to isolate the degree to which random error alone (and not bias) causes overestimation in individual meta-analyses. The sole effect of random error on the meta-analyzed intervention effect can, however, be accurately evaluated via simulation.

To assess the degree and likelihood with which imprecision causes overestimation of intervention effects at various stages of a meta-analysis, we undertook a simulation study. We measured the probability of observing relative risk reduction estimates that could potentially represent important overestimations after every 500 or 200 patients and for every 50 or 20 events (depending on the simulation scenario). We explored how well conventional information size requirements protected against overestimation by comparing these with the number of patients and events required for reducing the probability of overestimation to ‘acceptable levels’ (i.e., 10%, 5%, or 1%). Our simulations cover a comprehensive array of scenarios that approximate common meta-analysis data sets and our tables and figures may readily aid systematic review authors in assessing the risk of overestimation due to random error in their specific meta-analysis.

## Methods

### Simulation framework

We simulated binary meta-analysis data sets using a DerSimonian-Laird random-effects model framework [3], [18], [19]. The statistical formulation for the random-effects model as well as the formula for the DerSimonian-Laird estimator for the between-trial variance are presented in the supporting information (Appendix S1). We simulated meta-analysis scenarios based on assumed distributions and fixed, chosen values for the trial specific variables: the trial sample sizes, the control group risks, the ‘true’ intervention effect, and the degree of heterogeneity. We used two trial sample size distributions: one based on a survey of the Cochrane Heart Group meta-analyses on mortality (Table S1, S2) and one based on our subjective assessment of what constitutes a ‘common’ meta-analysis scenario. We used four different uniform distributions to sample the control group risk: 1% to 5% (representing ‘low’ control group risk), 5% to 15% (representing ‘moderately low’), 15% to 40% (representing ‘moderate’), and 40% to 80% (representing ‘high’). We used three different values of the between-trial variance (referred to as τ^2^ in the supporting information - Appendix S1) of the log relative risk to simulate different degrees of heterogeneity: 0.05, 0.15, and 0.25. Because our study objective was to investigate various aspects of overestimation of intervention effects, we used relative risk reduction (RRR) = 0% (no effect) and RRR = 10% (small but possibly unimportant effect) as the ‘true’ underlying intervention effects. In-depth rationale for the choice of the performed simulation scenarios is provided in appendix S2 in the supporting information. Further, the technical details of our simulation approach are described in detail in Appendix S2 in the supporting information.

**Figure 1:**
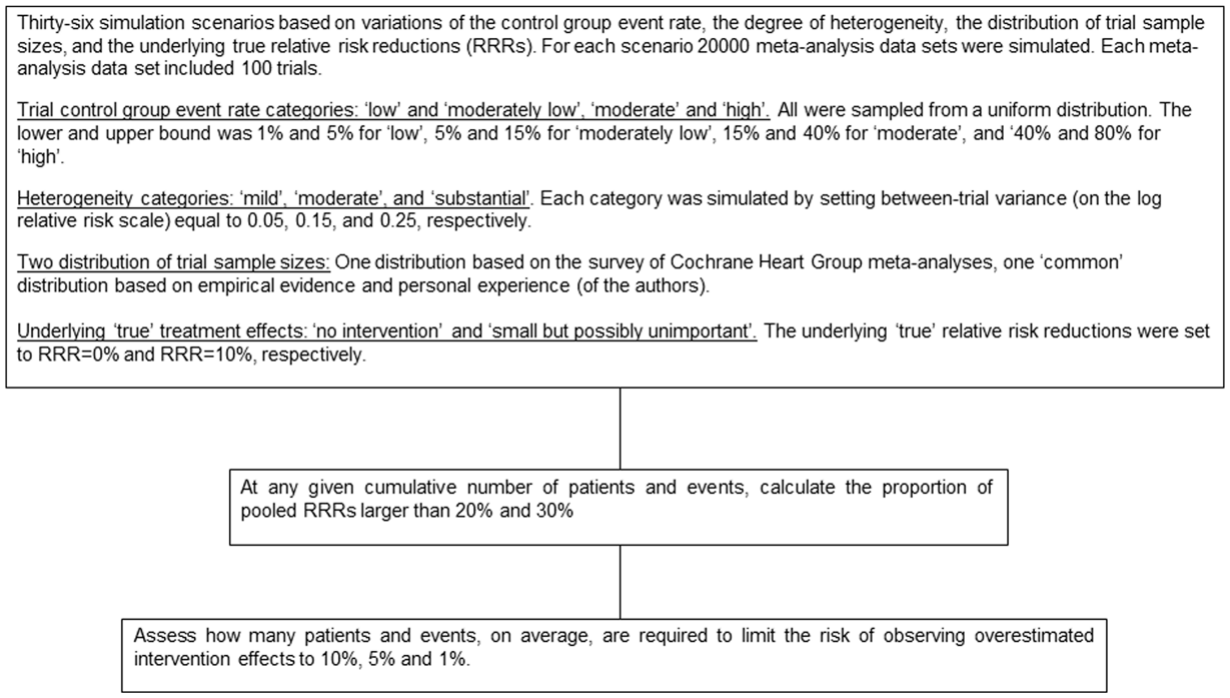
Flowchart of simulations and analyses. Simulation scenarios that included combinations of Cochrane Heart Group survey based trial sample size distribution and either ‘moderate’ or ‘high’ control group risks were not performed.

For each scenario, we simulated 20,000 meta-analysis data sets, and for each simulated meta-analysis data set, we simulated 100 trials. Although meta-analysis data sets including this many trials are uncommon in practice, we were interested in estimating the risk of overestimation both in common as well as uncommon meta-analysis scenarios. Simulating 100 trials for each meta-analysis data set allowed us to accurately estimate the risk of overestimation regardless of the cumulative number of patients and events. Figure 1 presents a flowchart of the simulation and analysis structure.

### The optimal information size

The optimal information size, OIS, for a binary outcome meta-analysis (also referred to as the required information size) is calculated as
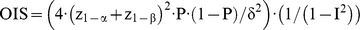
Where z_1-α_ and z_1-β_ are the (1-α)th and (1-β)th percentiles from the standard normal distribution, P is the average of the control group risk, P_C_, and intervention group risk, P_E_, δ is the difference between P_C_ and P_E_, and I^2^ is the popular (heterogeneity) measure for the proportion variation in a meta-analysis explained by differences between trials rather than sampling error. (Note, I^2^ is typically reported as a percentage (e.g., I^2^ = 35%), but in the OIS formula above, I^2^ is a proportion (e.g., I^2^ = 0.35)). The OIS provides the required number of patients in a meta-analysis to ensure that the maximum type I error is no larger than α and the maximum type II error is no larger than β when testing for statistical significance. The OIS can be converted to the required number of events by multiplying the required number of patients by P (assuming an approximately equal number of patients in the two groups).

## Analysis

For each simulation scenario of 20,000 cumulative meta-analyses data sets, we recorded the DerSimonian-Laird random-effects model cumulative meta-analyzed RRR (1 minus the meta-analyzed relative risk), the cumulative number of patients, and the cumulative number of events after each included trial. For each simulation set (i.e., true RRR = 0% and true RRR = 10%), we judged that RRR estimates larger than 20% and 30% could potentially represent important overestimates. At any given cumulative number of patients and events, we therefore calculated the proportion of simulated meta-analysis RRR that were larger than these thresholds.

We assessed the degree and likelihood of overestimation at various stages of a meta-analysis. For each scenario, we plotted the proportion of overestimates (according to the two thresholds) in relation to the cumulative number of patients and events. For each plot, we divided the cumulative number of patients into intervals of 500 or 200 (depending on the scenario), and the cumulative number of events into intervals of 50 or 20 (depending on the scenario).

We assessed how many patients and events were required to reduce the proportion of overestimates to acceptable levels, according to the two thresholds. We calculated the number of patients and events required to limit the probability of overestimation (according to the two thresholds) by 10%, 5%, and 1% - each of which could potentially constitute an ‘acceptable’ risk of overestimation.

We assessed the extent to which conventional information size requirements protect against overestimation. We calculated the optimal information sizes based on α = 5% and β = 20%, 10%, or 5%, with assumed control group risks set to the averages of the four control group risks distributions used in the simulation (i.e., P_C_ = 3.0%, P_C_ = 10.0%, P_C_ = 27.5%, or P_C_ = 60.0%), powered to detect intervention effects of RRR = 30% or RRR = 20%, and with heterogeneity corrections of I^2^ = 0.00, I^2^ = 0.25, or I^2^ = 0.50 (corresponding to I^2^ = 0%, I^2^ = 25%, and I^2^ = 50%). In total, 72 OIS estimates were calculated. We then compared the calculated information size requirements with the simulation results by matching OIS estimates with the scenarios where the underlying assumptions were similar. For example, the estimated probabilities of overestimation from the simulation based on a control group risk between 5% and 15% and tau^2^ = 0.15 was compared to the information size requirements based on an assumption of a 10% control group risk and 25% heterogeneity (I^2^ = 0.25 = 25%). For the comparison of information size requirements and simulation results, we post hoc created three categories for the ‘acceptability’ of the risk of overestimation: ‘good’, ‘very good’, and ‘excellent’. We defined ‘good’ acceptability as the situation where the probability of observing an RRR>20% was smaller than 10% and the probability of observing an RRR>30% was smaller than 5%. We defined ‘very good’ acceptability as the situation where the probability of observing an RRR>20% was smaller than 5% and the probability of observing an RRR>30% smaller than 1%. Lastly, we defined ‘excellent’ acceptability as the situation where the probability of observing an RRR>20% was smaller than 1%.

Of note, we did not record the probability of underestimation (i.e., we took a one-sided approach). Thus, 50% is the maximum observable probability of overestimation of intervention effects, and our results should be interpreted accordingly.

## Results

In most scenarios, the probability of overestimation was higher than 25% when the number of patients (or events) was small, but subsequently decreased exponentially (the x-axis is log scaled in Figure 2, and in Figures S1, S2, S3, S4, S5, S6, S7, S8, S9, S10, S11, S12).

**Figure 2:**
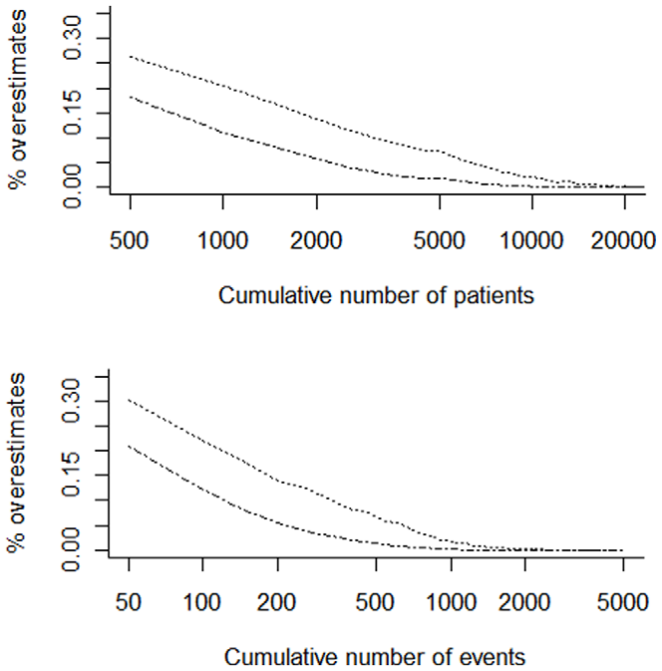
Presents the proportions of pooled intervention effects overestimating the relative risk reduction with 30% (– ▪ – ▪) and 20% (•••••••••) in the scenario with no underlying intervention effect (i.e., RRR = 0%), the trial sample size distribution is based on the Cochrane Heart Group survey, the control group risk is moderate (i.e., drawn from a uniform distribution between 5% and 15%) and the heterogeneity is moderate (i.e., τ^2^ = 0.15). The proportion of pooled intervention effect estimates (the risk of overestimation) are plotted in relation to the cumulative number of patients (upper plot) and events (lower plot).

Figure 2 presents the probability of overestimation in relation to the cumulative number of patients and events for a selected simulation scenario: no true intervention effect (RRR = 0%), moderate control group event risk (uniform distribution from 5% to 15%), and moderate heterogeneity (between-trial variance τ^2^ = 0.15), and distribution of trials sizes based on our survey of the Cochrane Heart Group meta-analyses. Figures S1, S2, S3, S4, S5, S6, S7, S8, S9, S10, S11, and S12 present the probability of overestimation in relation to the cumulative number of patients and events for all simulation scenarios.

The number of patients and events required for the probability of overestimation to drop below 10%, 5%, and 1% in the simulated scenarios are presented in Table 1, and Tables S3 and S4. Table 1 presents the scenarios where the distribution of trial sample sizes were based on our survey of the Cochrane Heart Group meta-analyses, and Tables S3 and S4 present the scenarios where the distribution of trial sample sizes were based on our assessment of what we subjectively assessed constituted a ‘common’ meta-analysis scenario.

**Table 1:** Presents the required number of patients and events for the probability of overestimation to drop below 10%, 5% and 1%, in scenarios where the control group risk is ‘low’ or ‘moderately low’ and where the distribution of trial sample sizes is based on a survey of 23 Cochrane Heart Group meta-analyses on mortality.

Scenario parameters				Number of patients required for the probability of overestimation to drop below			Number of events required for the probability of overestimation to drop below		
*True effect*	*Overestimation*	*PC*	*τ^2^*	10%	5%	1%	10%	5%	1%
*RRR = 0%*	*RRR>30%*	1%–5%	0.05	2000	3500	8000	100	150	300
			0.15	2500	4500	10500	100	150	350
			0.25	3000	5500	11500	150	200	350
		5%–15%	0.05	1000	1500	3500	100	150	350
			0.15	1500	2500	6500	150	250	600
			0.25	1500	3500	8000	200	350	750
	*RRR>20%*	1%–5%	0.05	5500	9000	19500	200	300	600
			0.15	6500	10500	21500	250	350	650
			0.25	6500	11500	23000	250	350	700
		5%–15%	0.05	2500	4000	9000	200	400	850
			0.15	3000	6500	13000	350	600	1250
			0.25	4500	8000	16500	450	750	1650
*RRR = 10%*	*RRR>30%*	1%–5%	0.05	4000	7000	14500	150	250	450
			0.15	5500	9000	18000	200	300	550
			0.25	5500	9000	18500	200	300	550
		5%–15%	0.05	2000	3000	7500	200	300	700
			0.15	2500	5500	11000	250	450	1000
			0.25	3500	7000	14000	350	600	1250
	*RRR>20%*	1%–5%	0.05	16500	26500	>50000	500	800	1650
			0.15	15000	25000	>50000	500	800	1500
			0.25	14500	24000	>50000	450	750	1450
		5%–15%	0.05	7500	13500	26500	700	1250	2500
			0.15	10000	17000	37000	950	1600	3400
			0.25	12000	19500	40000	1150	1850	3750

The number of patients and events required to limit the risk of overestimation depended on the threshold for overestimation (i.e., RRR = 20% or RRR = 30%) and all the considered simulation components: relative risk reduction, control group risk, heterogeneity, and trial size distribution. The larger the overestimation (i.e., the larger the difference between the meta-analyzed and the true RRR), the smaller the number of patients and events required to limit the risk of overestimation. A larger number of patients were required to limit the risk of overestimation in the scenarios where the control group risk was low. Conversely, a smaller number of events were required to limit the risk of overestimation in the scenarios with control group risk was low. The number of patients and events required to limit the risk of overestimation was generally smaller in scenarios when heterogeneity was set at the lowest level (τ^2^ = 0.05) than when it was set to the highest level (τ^2^ = 0.25). In contrast, in scenarios with the ‘common’ trial size distribution and with low control group risks (1–5%), the number of patients and events required was higher when heterogeneity was lowest. This reversed pattern was also observed in a few other isolated scenarios.

Table 2 presents the calculated optimal information size for 72 different settings (see analysis section for more detail). Table 3 and 4 present of the number of patients and events required to limit the risk of overestimation, grouped by control group risk and distribution of trial sample size. The calculated OIS are included in these tables for comparison. In scenarios with low control group risk (1%–5%), the risk of overestimation generally reached very good or excellent acceptability before reaching optimal information sizes (based on 80% power or 90% power). In scenarios with moderately low control group risk (5% to 15%), good acceptability was commonly reached before or close to the OIS based on 80% power, whereas very good and sometimes excellent acceptability was reached before the OIS based on 90% power or 95% power. In scenarios with moderate control group risk (15% to 40%), good acceptability was reached before the OIS based on 80% power and very good acceptability was usually reached before the OIS based on 95% power. In scenarios with high control group risk (40% to 80%), good acceptable was often (but not always) reached before the OIS based on 95% power. Some exceptions were observed in all of the above generalizations when the heterogeneity was large (i.e., τ^2^ = 0.25).

**Table 2:** Presents the calculated optimal information size (OIS) to detect RRR = 30% and RRR = 20% respectively depending on the underlying assumed control group risk (*PC*), a desired type I error of 5%, variations of the desired type II error (*β* = 20%, 10%, or 5%) and the anticipated degree of heterogeneity.

Scenario parameters			OIS (required number of patients)			OIS (required number of events)		
*Assumed effect*	*PC*	*I^2^*	β = 20%	β = 10%	β = 5%	β = 20%	β = 10%	β = 5%
*RRR = 30%*	3%	0%	9600	13000	16000	250	350	400
		25%	13000	17000	21500	350	450	550
		50%	19500	26000	32000	500	650	800
*RRR = 20%*		0%	23000	30500	38000	600	850	1000
		25%	30500	41000	51000	850	1100	1350
		50%	46000	61000	76000	1250	1650	2050
*RRR = 30%*	10%	0%	2700	3600	4500	250	300	400
		25%	3500	5000	6000	300	400	500
		50%	5500	7500	9000	450	600	750
*RRR = 20%*		0%	6500	8500	10500	600	800	1000
		25%	8500	11500	14000	750	1000	1300
		50%	13000	17000	21500	1150	1550	1900
*RRR = 30%*	27.5%	0%	900	1100	1400	200	300	350
		25%	1100	1500	1800	250	350	450
		50%	1700	2200	2700	400	550	650
*RRR = 20%*		0%	1900	2600	3200	500	650	800
		25%	2600	3500	4300	650	850	1050
		50%	3900	5200	6400	950	1300	1600
*RRR = 30%*	60%	0%	200	300	400	150	200	200
		25%	300	400	500	200	250	300
		50%	500	600	800	250	350	400
*RRR = 20%*		0%	500	700	900	300	400	500
		25%	700	1000	1200	400	550	650
		50%	1100	1500	1800	600	800	950

The required number of events have been rounded up to the nearest number divisible by 50. The required number of patients have been rounded up to the nearest number divisible by 1000 when PC = 3% and PC = 10% and to the nearest number divisible by 100 when PC = 27.5% and PC = 60%.

**Table 3:** Presents the comparison of the optimal information size to demonstrate a relevant intervention effect with the required number of patients and events to limit the risk of overestimation in simulation scenarios where the distribution of trial sample sizes was based on survey of Cochrane Heart Group meta-analyses.

Simulation					Optimal Information Size (OIS)				
PC	Overestimation	Acceptability	Patients	Events	PC	RRR	Power	Patients	Events
1%–5%	RRR>30%	Good	3500–5500	150–200	3%	30%	80%	10000–20000	250–500
		Very Good	7000–11500	250–350			90%	13000–26000	350–650
		Excellent	14500–18500	450–550			95%	16000–32000	400–800
	RRR>20%	Good	10000–15000	400–500		20%	80%	23000–46000	600–1250
		Very Good	20000–25000	600–800			90%	30000–61000	850–1650
		Excellent	>50000	1400–1600			95%	38000–76000	1000–2050
5%–15%	RRR>30%	Good	2000–4000	200–300	3%	30%	80%	3000–5500	250–450
		Very Good	3000–8000	300–700			90%	3500–7500	300–600
		Excellent	7000–14000	700–1200			95%	4500–9000	400–750
	RRR>20%	Good	7000–12000	600–1200		20%	80%	6500–13000	600–1150
		Very Good	9000–19000	1250–1850			90%	8500–17000	800–1600
		Excellent	26000–40000	2500–2800			95%	10500–21000	1000–1900

**Table 4:** Presents the comparison of the optimal information size (OIS) to demonstrate a relevant intervention effect with the required number of patients and events to limit the risk of overestimation in simulation scenarios where the distribution of trial sample sizes was based on survey of Cochrane Heart Group meta-analyses.

Simulation					Optimal information size				
PC	Overestimation	Acceptability	Patients	Events	PC	RRR	Power	Patients	Events
1%–5%	RRR>30%	Good	2500	100	3%	30%	80%	10–20000	250–500
		Very Good	3500–4500	150–200			90%	13–26000	350–650
		Excellent	6000–7500	200–250			95%	16–32000	400–800
	RRR>20%	Good	4000–7500	150–250		20%	80%	23–46000	600–1250
		Very Good	7000–11000	250–400			90%	30–61000	850–1650
		Excellent	14000–19000	350–600			95%	38–76000	1000–2050
5%–15%	RRR>30%	Good	1500	100–150	10%	30%	80%	3000–5500	250–450
		Very Good	2000–3000	200–250			90%	3500–7500	300–600
		Excellent	3500–4500	350–450			95%	4500–9000	400–750
	RRR>20%	Good	2500–3500	250–350		20%	80%	6500–13000	600–1150
		Very Good	4500–5500	450–600			90%	8500–17000	800–1600
		Excellent	11000–12000	900–1150			95%	10500–21000	1000–1900
15%–40%	RRR>30%	Good	500–2500	150–700	27.5%	30%	80%	800–1700	200–400
		Very Good	1400–6200	400–1700			90%	1100–2200	300–550
		Excellent	4000–12000	1000–3000			95%	1400–2700	350–650
	RRR>20%	Good	1000–3000	300–850		20%	80%	1900–3600	500–950
		Very Good	2100–5400	550–1350			90%	2600–5200	650–1300
		Excellent	6200–11400	1500–2300			95%	3200–6400	800–1600
40%–80%	RRR>30%	Good	200–1000	150–500	60%	30%	80%	200–500	150–250
		Very Good	600–1800	300–1000			90%	300–600	200–350
		Excellent	1100–3200	600–1600			95%	400–800	200–400
	RRR>20%	Good	700–3400	350–1950		20%	80%	500–1100	300–600
		Very Good	1400–5800	750–3500			90%	700–1500	400–800
		Excellent	4000–11000	2000–5000			95%	900–1800	500–950

## Discussion

Our simulations provide valuable insight on the risk of overestimation of intervention effects in meta-analysis due to random errors over time. The risk of observing overestimated intervention effects due to random error at ‘early’ stages of a meta-analysis is substantial. The number of patients and events required to limit this risk depend considerably on each of the components considered in our simulation study: the degree of overestimation that is considered to be important, the underlying true effect, the control group risk, the degree of heterogeneity, and the distribution of trial sample sizes. However, the comparison of our simulation results with the approximately corresponding information size requirements demonstrated that upon reaching the OIS in a meta-analysis, one can be relatively confident that the intervention effect is not overestimated due to random error.

Our study comes with several strengths and limitations. Our simulations covered a wide spectrum of meta-analysis scenarios which we believe occur frequently in the systematic review literature. Our simulation results therefore have good generalizability to meta-analysis in practice. While the spectrum of scenarios covered in our simulations is not as extensive as seen in some previous meta-analysis simulation studies, adding additional scenarios to the current study would likely increase the complexity and hamper the interpretability of our findings. We believe the chosen spectrum of our simulations constitute a close to optimal balance between interpretability and generalizability.

Our simulation study is the first of its kind to contrast the risk of overestimation of intervention effects due to random errors with information size requirements. The statistical purpose of calculating the OIS is to gain control over the risk of obtaining a false positive finding (type I error) and a false negative finding (type II error). Extending this purpose, authors have previously considered information size requirements as a means of gaining control over the risk of overestimation [2], [20]. Our simulation study is the first to explore the validity of this theoretical claim. However, we only investigated the extent to which information size requirements protect against overestimation when the underlying assumptions (e.g., a priori assumed RRR and control group risk) matched the parameter settings in a given simulation scenario (e.g., the assumed control group risk for the optimal information size was set to 10% when the control group risk in the simulation was sampled from a uniform distribution between 5% and 15%). That is, our findings hold for information sizes that have been calculated using the appropriate assumptions for a given scenario. In reality, it can be difficult to know which assumptions are most appropriate when doing information size calculations for a meta-analysis. The implications of employing overly lenient or conservative a priori assumptions for the OIS are, theoretically, relatively straightforward. Lenient assumptions (e.g., β = 20% and RRR = 0.35) will results in relatively small information size requirements, and thus, an inappropriately high degree of confidence that the estimated intervention effect can be trusted (i.e., is not an overestimate). Conversely, conservative assumptions (e.g., α = 0.1% and RRR = 0.10) have the potential to remove confidence about an intervention effect estimate, even if the intervention effect estimate is in fact reliable.

We mentioned in the introduction that various types of bias (e.g., methodological bias or publication bias) may also be important causes of overestimation of intervention effects [3], [13]. We did not attempt to include any biases in our simulations. It is likely that when bias is present in a meta-analysis, a larger number of patients and events will be required to limit the risk of overestimation. In some cases, bias may limit the reliability of the size of the intervention estimate independent of how large the meta-analysis is.

Another limitation of our simulations is that the underlying true trial effects were sampled as random effects. This approach does not consider the possibility that the magnitude of trial effects to some extent may depend on time. For example, the first series of trials in a meta-analysis compared to the later trials may generally recruit a broader or narrower population, use shorter or longer follow-up, or administer higher or lower doses of a drug. Depending on the effect such time dependencies have on the evolution of the meta-analyzed intervention effect, the number of patients and events required to limit overestimation may be either larger or smaller than our results indicate.

Our simulation results are consistent with the results of previous empirical studies. More specifically, the pooled intervention effect estimates tend to fluctuate considerably when the number of patients and events are sparse, thus creating a high risk of overestimation [4]–[6], [12]. Ioannidis and Lau previously investigated convergence of intervention effects in two fields, interventions in pregnancy and perinatal medicine and management of myocardial infarction. They found that more than 10,000 patients were generally required to relieve uncertainty about subsequent changes in meta-analyzed intervention effects [4]. Trikalinos et al. performed a similar study on interventions within the field of mental health and found that only 2000 patients were required to relieve uncertainty about subsequent changes in meta-analyzed intervention effects [5]. The meta-analyses considered by Ioannidis and Lau were similar to many of our simulated scenarios where the control group risk was ‘low’ and ‘moderately low’. The meta-analyses considered by Trikalinos et al. were similar to many of our simulated scenarios where the control group risk was ‘moderate’ or ‘high’.

The results of our simulation study have several implications. First, they underscore the need for information size requirements in all meta-analyses. Second, they illustrate the dangers of relying on intervention effect estimates before the OIS is reached (or is close to being reached), even when the presence of bias is unlikely. The figures in the supporting information provide meta-analysts with an opportunity to check the approximate risk of overestimation due to random error in their meta-analyses.

Two key inferential measures in a meta-analysis are the p-value and the 95% confidence interval associated with the estimated intervention effect. We wish to offer additional caution in interpreting meta-analyzed intervention effect estimates in the face of limited evidence. Large effect estimates (true or false) do not require high precision to reach conventional statistical significance (i.e., p≤0.05). As demonstrated in empirical studies, early large intervention effects are likely to dissipate and early statistically significant meta-analyses are likely to be false positives [4]–[6], [12], [21]. Therefore, when observing a large statistically significant intervention effect estimate (e.g., RRR>30%) in a meta-analysis including a limited number of patients and events, one should always consider whether the meta-analysis, with the same precision, would have been statistically significant had the observed intervention effect been moderate or small. Chances are it would not. By the same token, one should always consider what values the confidence interval would have included had the effect estimate been moderate or small.

Even if an ‘early’ large intervention effect estimate is not supported by formal statistical significance, the situation may still be problematic. Large intervention effects will encourage clinical trial investigators to conduct further trials, and systematic review authors to perform regular updates of the meta-analysis until it either reaches statistical significance or the early trend has been definitively refuted. Updates of meta-analysis cause multiplicity due to repeated significance testing – a conduct which has been documented as highly problematic [6], [10], [22]–[24]. In particular, multiple testing increases the risk of observing a falsely significant result before the optimal information size has been surpassed. This may very well happen at a point where the risk of overestimation is still substantial. Moreover, in the face of repeated significance testing, confidence intervals suffer from reduced coverage, and thus an increased risk of precluding the ‘true’ intervention effect. Multiplicity due to repeated significance testing in meta-analysis can be accounted for by employing sequential testing procedure like the O'Brien-Fleming group sequential boundaries (i.e., adjusted thresholds for statistical significance) and adjusted confidence intervals can be constructed accordingly. Evidence suggests that these techniques provide adequate protection against false positives [6], [8], [14], [22]. Given that such adjusted significance thresholds and the corresponding adjusted confidence intervals are tied to the calculated information size requirement, and given that information size criteria seem to provide adequate protection against ‘early’ overestimation, it seems reasonable to believe that adjusted significance thresholds and confidence intervals are appropriate inferential measures for interpreting early intervention effect estimates in meta-analysis.

In conclusion, the risk of overestimated intervention effects in meta-analysis due to random error is often substantial in the face of a limited number of patients and events. Insisting that a meta-analysis meets a reasonable OIS will ensure an acceptably low risk of observing an overestimated intervention effect due to random errors.

## Supporting Information

Figure S1 **Presents the proportions of pooled intervention effects exceeding a relative risk reduction of 30% (– ▪ – ▪) and 20% (•••••••••) when there is no underlying intervention effect (i.e., RRR = 0%), and where the distribution trial sample sizes are based on the survey of 23 Cochrane Heart Group meta-analyses.** The proportions are plotted in relation to the cumulative number of patients. The upper three plots present the results from the simulated scenarios where the underlying ‘true’ trial control group risks are drawn from a uniform distribution between 1% and 5% (‘low’ risk), and the lower three plots present the results from the simulated they are drawn from a uniform distribution between 5% and 15% (‘moderately low’ risk). The two left plots present results from scenarios with ‘mild’ heterogeneity (τ^2^ = 0.05), the middle two results from scenarios with moderate heterogeneity(τ^2^ = 0.15), and the two right plots results from scenarios with substantial heterogeneity (τ^2^ = 0.25).
(TIFF)

Figure S2 **Presents the proportions of pooled intervention effects exceeding a relative risk reduction of 30% (– ▪ – ▪) and 20% (•••••••••) when there is no underlying intervention effect (i.e., RRR = 0%), and where the distribution trial sample sizes are based on the survey of 23 Cochrane Heart Group meta-analyses.** The proportions are plotted in relation to the cumulative number of events. The upper three plots present the results from the simulated scenarios where the underlying ‘true’ trial control group risks are drawn from a uniform distribution between 1% and 5% (‘low’ risk), and the lower three plots present the results from the simulated they are drawn from a uniform distribution between 5% and 15% (‘moderately low’ risk). The two left plots present results from scenarios with ‘mild’ heterogeneity (τ^2^ = 0.05), the middle two results from scenarios with moderate heterogeneity(τ^2^ = 0.15), and the two right plots results from scenarios with substantial heterogeneity (τ^2^ = 0.25).
(TIFF)

Figure S3 **Presents the proportions of pooled intervention effects exceeding a relative risk reduction of 30% (– ▪ – ▪) and 20% (•••••••••) when there is a small but potentially important intervention effect (i.e., RRR = 10%), and where the distribution trial sample sizes are based on the survey of 23 Cochrane Heart Group meta-analyses.** The proportions are plotted in relation to the cumulative number of patients. The upper three plots present the results from the simulated scenarios where the underlying ‘true’ trial control group risks are drawn from a uniform distribution between 1% and 5% (‘low’ risk), and the lower three plots present the results from the simulated they are drawn from a uniform distribution between 5% and 15% (‘moderately low’ risk). The two left plots present results from scenarios with ‘mild’ heterogeneity (τ^2^ = 0.05), the middle two results from scenarios with moderate heterogeneity (τ^2^ = 0.15), and the two right plots results from scenarios with substantial heterogeneity (τ^2^ = 0.25).
(TIFF)

Figure S4 **Presents the proportions of pooled intervention effects exceeding a relative risk reduction of 30% (– ▪ – ▪) and 20% (•••••••••) when there is small but potentially important intervention effect (i.e., RRR = 10%), and where the distribution trial sample sizes are based on the survey of 23 Cochrane Heart Group meta-analyses.** The proportions are plotted in relation to the cumulative number of events. The upper three plots present the results from the simulated scenarios where the underlying ‘true’ trial control group risks are drawn from a uniform distribution between 1% and 5% (‘low’ risk), and the lower three plots present the results from the simulated they are drawn from a uniform distribution between 5% and 15% (‘moderately low’ risk). The two left plots present results from scenarios with ‘mild’ heterogeneity (τ^2^ = 0.05), the middle two results from scenarios with moderate heterogeneity (τ^2^ = 0.15), and the two right plots results from scenarios with substantial heterogeneity (τ^2^ = 0.25).
(TIFF)

Figure S5 **Presents the proportions of pooled intervention effects exceeding a relative risk reduction of 30% (– ▪ – ▪) and 20% (•••••••••) when there is no underlying intervention effect (i.e., RRR = 0%), and where the distribution trial sample sizes are our assessment of what constitutes ‘common’ meta-analysis trial size distributions.** The proportions are plotted in relation to the cumulative number of patients. The upper three plots present the results from the simulated scenarios where the underlying ‘true’ trial control group risks are drawn from a uniform distribution between 1% and 5% (‘low’ risk), and the lower three plots present the results from the simulated they are drawn from a uniform distribution between 5% and 15% (‘moderately low’ risk). The two left plots present results from scenarios with ‘mild’ heterogeneity (τ^2^ = 0.05), the middle two results from scenarios with moderate heterogeneity (τ^2^ = 0.15), and the two right plots results from scenarios with substantial heterogeneity (τ^2^ = 0.25).
(TIFF)

Figure S6 **Presents the proportions of pooled intervention effects exceeding a relative risk reduction of 30% (– ▪ – ▪) and 20% (•••••••••) when there is no underlying intervention effect (i.e., RRR = 0%), and where the distribution trial sample sizes are our assessment of what constitutes ‘common’ meta-analysis trial size distributions.** The proportions are plotted in relation to the cumulative number of events. The upper three plots present the results from the simulated scenarios where the underlying ‘true’ trial control group risks are drawn from a uniform distribution between 1% and 5% (‘low’ risk), and the lower three plots present the results from the simulated they are drawn from a uniform distribution between 5% and 15% (‘moderately low’ risk). The two left plots present results from scenarios with ‘mild’ heterogeneity (τ^2^ = 0.05), the middle two results from scenarios with moderate heterogeneity(τ^2^ = 0.15), and the two right plots results from scenarios with substantial heterogeneity (τ^2^ = 0.25).
(TIFF)

Figure S7 **Presents the proportions of pooled intervention effects exceeding a relative risk reduction of 30% (– ▪ – ▪) and 20% (•••••••••) when there is small but potentially important intervention effect (i.e., RRR = 10%), and where the distribution trial sample sizes are our assessment of what constitutes ‘common’ meta-analysis trial size distributions.** The proportions are plotted in relation to the cumulative number of patients. The upper three plots present the results from the simulated scenarios where the underlying ‘true’ trial control group risks are drawn from a uniform distribution between 1% and 5% (‘low’ risk), and the lower three plots present the results from the simulated they are drawn from a uniform distribution between 5% and 15% (‘moderately low’ risk). The two left plots present results from scenarios with ‘mild’ heterogeneity (τ^2^ = 0.05), the middle two results from scenarios with moderate heterogeneity (τ^2^ = 0.15), and the two right plots results from scenarios with substantial heterogeneity (τ^2^ = 0.25).
(TIFF)

Figure S8 **Presents the proportions of pooled intervention effects exceeding a relative risk reduction of 30% (– ▪ – ▪) and 20% (•••••••••) when there is small but potentially important intervention effect (i.e., RRR = 10%), and where the distribution trial sample sizes are our assessment of what constitutes ‘common’ meta-analysis trial size distributions.** The proportions are plotted in relation to the cumulative number of events. The upper three plots present the results from the simulated scenarios where the underlying ‘true’ trial control group risks are drawn from a uniform distribution between 1% and 5% (‘low’ risk), and the lower three plots present the results from the simulated they are drawn from a uniform distribution between 5% and 15% (‘moderately low’ risk). The two left plots present results from scenarios with ‘mild’ heterogeneity (τ^2^ = 0.05), the middle two results from scenarios with moderate heterogeneity (τ^2^ = 0.15), and the two right plots results from scenarios with substantial heterogeneity (τ^2^ = 0.25).
(TIFF)

Figure S9 **Presents the proportions of pooled intervention effects exceeding a relative risk reduction of 30% (– ▪ – ▪) and 20% (•••••••••) when there is no underlying intervention effect (i.e., RRR = 0%), and where the distribution trial sample sizes are our assessment of what constitutes ‘common’ meta-analysis trial size distributions.** The proportions are plotted in relation to the cumulative number of patients. The upper three plots present the results from the simulated scenarios where the underlying ‘true’ trial control group risks are drawn from a uniform distribution between 15% and 40% (‘moderate’ risk), and the lower three plots present the results from the simulated they are drawn from a uniform distribution between 40% and 80% (‘high’ risk). The two left plots present results from scenarios with ‘mild’ heterogeneity (τ^2^ = 0.05), the middle two results from scenarios with moderate heterogeneity (τ^2^ = 0.15), and the two right plots results from scenarios with substantial heterogeneity (τ^2^ = 0.25).
(TIFF)

Figure S10 **Presents the proportions of pooled intervention effects exceeding a relative risk reduction of 30% (– ▪ – ▪) and 20% (•••••••••) when there is no underlying intervention effect (i.e., RRR = 0%), and where the distribution trial sample sizes are our assessment of what constitutes ‘common’ meta-analysis trial size distributions.** The proportions are plotted in relation to the cumulative number of events. The upper three plots present the results from the simulated scenarios where the underlying ‘true’ trial control group risks are drawn from a uniform distribution between 15% and 40% (‘moderate’ risk), and the lower three plots present the results from the simulated they are drawn from a uniform distribution between 40% and 80% (‘high’ risk). The two left plots present results from scenarios with ‘mild’ heterogeneity (τ^2^ = 0.05), the middle two results from scenarios with moderate heterogeneity (τ^2^ = 0.15), and the two right plots results from scenarios with substantial heterogeneity (τ^2^ = 0.25).
(TIFF)

Figure S11 **Presents the proportions of pooled intervention effects exceeding a relative risk reduction of 30% (– ▪ – ▪) and 20% (•••••••••) when there is small but potentially important intervention effect (i.e., RRR = 10%), and where the distribution trial sample sizes are our assessment of what constitutes ‘common’ meta-analysis trial size distributions.** The proportions are plotted in relation to the cumulative number of patients. The upper three plots present the results from the simulated scenarios where the underlying ‘true’ trial control group risks are drawn from a uniform distribution between 15% and 40% (‘moderate’ risk), and the lower three plots present the results from the simulated they are drawn from a uniform distribution between 40% and 80% (‘high’ risk). The two left plots present results from scenarios with ‘mild’ heterogeneity (τ^2^ = 0.05), the middle two results from scenarios with moderate heterogeneity (τ^2^ = 0.15), and the two right plots results from scenarios with substantial heterogeneity (τ^2^ = 0.25).
(TIFF)

Figure S12 **Presents the proportions of pooled intervention effects exceeding a relative risk reduction of 30% (– ▪ – ▪) and 20% (•••••••••) when there is small but potentially important intervention effect (i.e., RRR = 10%), and where the distribution trial sample sizes are our assessment of what constitutes ‘common’ meta-analysis trial size distributions.** The proportions are plotted in relation to the cumulative number of events. The upper three plots present the results from the simulated scenarios where the underlying ‘true’ trial control group risks are drawn from a uniform distribution between 15% and 40% (‘moderate’ risk), and the lower three plots present the results from the simulated they are drawn from a uniform distribution between 40% and 80% (‘high’ risk). The two left plots present results from scenarios with ‘mild’ heterogeneity (τ^2^ = 0.05), the middle two results from scenarios with moderate heterogeneity (τ^2^ = 0.15), and the two right plots results from scenarios with substantial heterogeneity (τ^2^ = 0.25).
(TIFF)

Figure S13 **Histogram of trial sample sizes in the surveyed Cochrane heart group meta-analyses.**
(TIF)

Table S1 **Presents the recorded meta-analysis and trial characteristics from the survey of Cochrane Heart Group mortality meta-analyses.** The column labeled ‘Quartile’ contains the 25^th^ to 75^th^ percentile interval. The columns labeled ‘Spectrum’ contains the minimum and maximum value observed. The last column contains the DerSimonian-Laird estimate of the between-trial variance (on the log relative risk scale).
(DOC)

Table S2 **Estimated proportions of trial sample sizes based on the survey of Cochrane Heart Group meta-analysis as well as proportions used in our simulations.**
(DOC)

Table S3 **Presents the required number of patients and events for the probability of overestimation to drop below 10%, 5% and 1%, in the simulation based on the sensitivity trial size distribution.**
(DOC)

Table S4 **Presents the required number of patients and events for the probability of overestimation to drop below 10%, 5% and 1%, in the simulation based on the sensitivity trial size distribution.**
(DOC)

Appendix S1 **Presents the conventional random-effects model meta-analysis setup and the DerSimonian-Laird random-effects model.**
(DOC)

Appendix S2 **Presents the complete simulation setup as well as the rationale for the choice of parameter distributions and fixed values.**
(DOC)
