# Supplementary figures and images for: The Number of Patients and Events Required to Limit the Risk of Overestimation of Intervention Effects in Meta-Analysis—A Simulation Study
Source: PLoS One. 2011 Oct 18;6(10):e25491. doi: 10.1371/journal.pone.0025491 (PMC3196500; doi:10.1371/journal.pone.0025491)

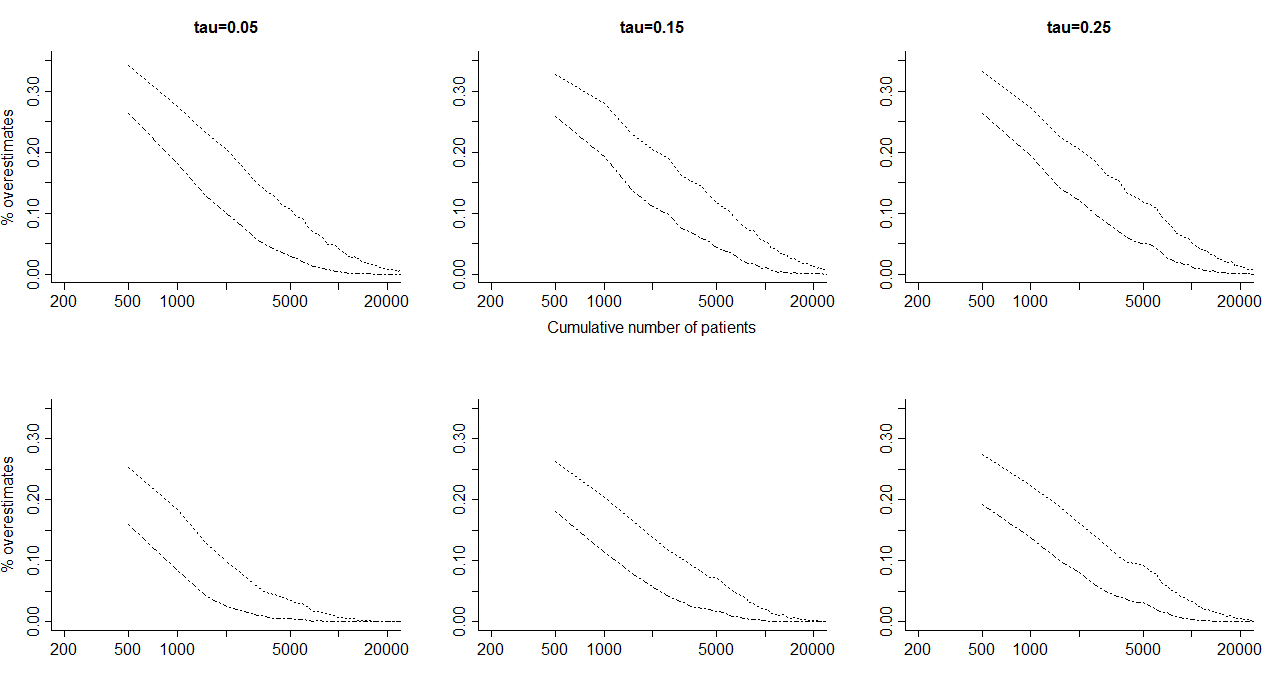

Supplement: Figure S1 — Presents the proportions of pooled intervention effects exceeding a relative risk reduction of 30% (– ▪ – ▪) and 20% (•••••••••) when there is no underlying intervention effect (i.e., RRR = 0%), and where the distribution trial sample sizes are based on the survey of 23 Cochrane Heart Group meta-analyses. The proportions are plotted in relation to the cumulative number of patients. The upper three plots present the results from the simulated scenarios where the underlying ‘true’ trial control group risks are drawn from a uniform distribution between 1% and 5% (‘low’ risk), and the lower three plots present the results from the simulated they are drawn from a uniform distribution between 5% and 15% (‘moderately low’ risk). The two left plots present results from scenarios with ‘mild’ heterogeneity (τ2 = 0.05), the middle two results from scenarios with moderate heterogeneity(τ2 = 0.15), and the two right plots results from scenarios with substantial heterogeneity (τ2 = 0.25). (TIFF) [file pone.0025491.s001.tif]

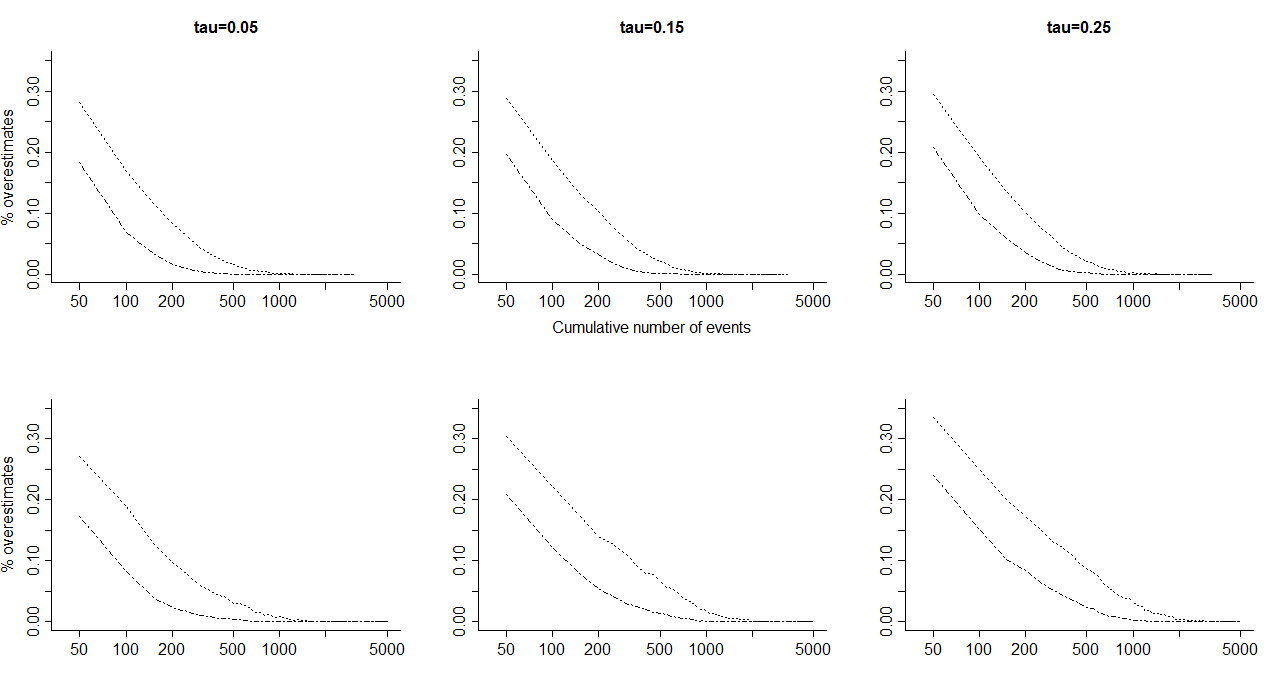

Supplement: Figure S2 — Presents the proportions of pooled intervention effects exceeding a relative risk reduction of 30% (– ▪ – ▪) and 20% (•••••••••) when there is no underlying intervention effect (i.e., RRR = 0%), and where the distribution trial sample sizes are based on the survey of 23 Cochrane Heart Group meta-analyses. The proportions are plotted in relation to the cumulative number of events. The upper three plots present the results from the simulated scenarios where the underlying ‘true’ trial control group risks are drawn from a uniform distribution between 1% and 5% (‘low’ risk), and the lower three plots present the results from the simulated they are drawn from a uniform distribution between 5% and 15% (‘moderately low’ risk). The two left plots present results from scenarios with ‘mild’ heterogeneity (τ2 = 0.05), the middle two results from scenarios with moderate heterogeneity(τ2 = 0.15), and the two right plots results from scenarios with substantial heterogeneity (τ2 = 0.25). (TIFF) [file pone.0025491.s002.tif]

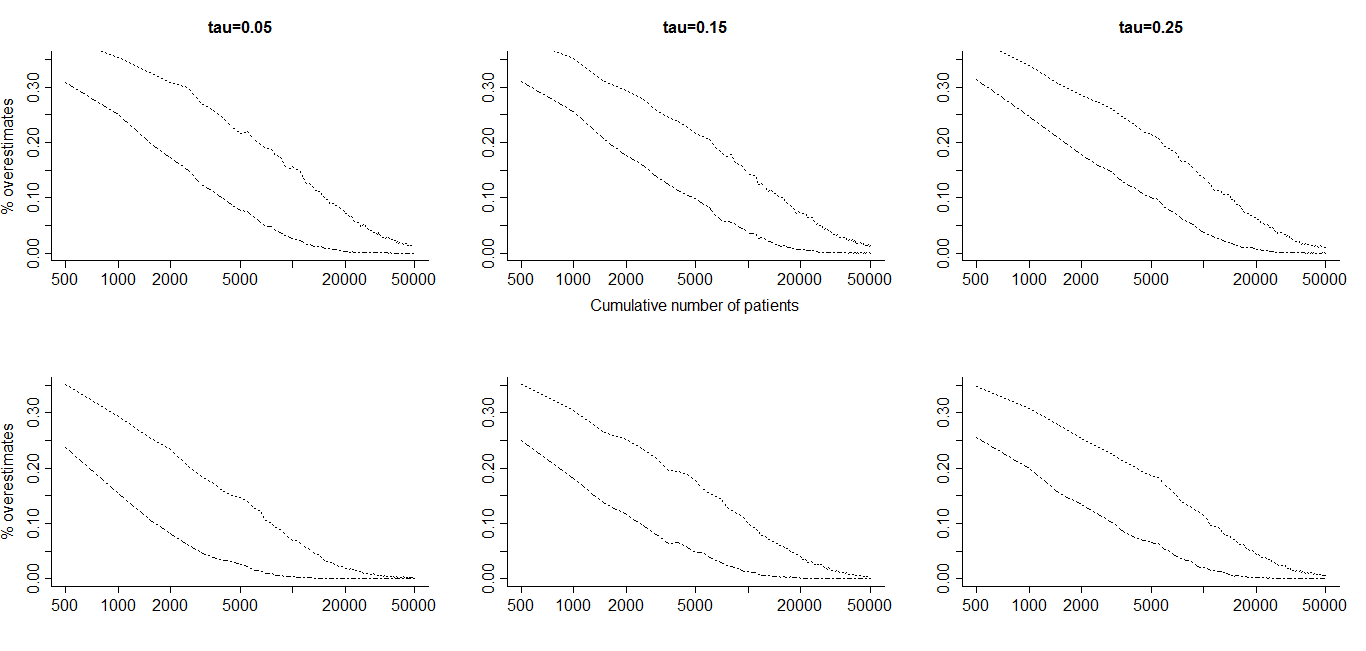

Supplement: Figure S3 — Presents the proportions of pooled intervention effects exceeding a relative risk reduction of 30% (– ▪ – ▪) and 20% (•••••••••) when there is a small but potentially important intervention effect (i.e., RRR = 10%), and where the distribution trial sample sizes are based on the survey of 23 Cochrane Heart Group meta-analyses. The proportions are plotted in relation to the cumulative number of patients. The upper three plots present the results from the simulated scenarios where the underlying ‘true’ trial control group risks are drawn from a uniform distribution between 1% and 5% (‘low’ risk), and the lower three plots present the results from the simulated they are drawn from a uniform distribution between 5% and 15% (‘moderately low’ risk). The two left plots present results from scenarios with ‘mild’ heterogeneity (τ2 = 0.05), the middle two results from scenarios with moderate heterogeneity (τ2 = 0.15), and the two right plots results from scenarios with substantial heterogeneity (τ2 = 0.25). (TIFF) [file pone.0025491.s003.tif]

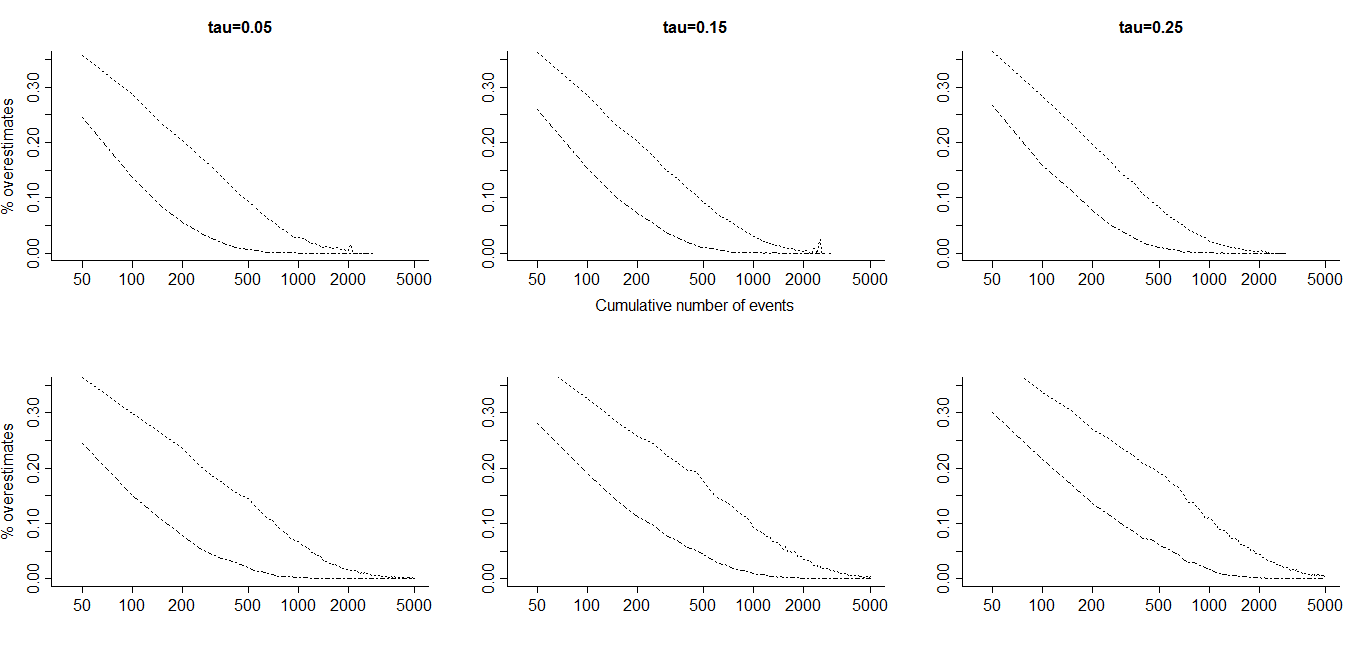

Supplement: Figure S4 — Presents the proportions of pooled intervention effects exceeding a relative risk reduction of 30% (– ▪ – ▪) and 20% (•••••••••) when there is small but potentially important intervention effect (i.e., RRR = 10%), and where the distribution trial sample sizes are based on the survey of 23 Cochrane Heart Group meta-analyses. The proportions are plotted in relation to the cumulative number of events. The upper three plots present the results from the simulated scenarios where the underlying ‘true’ trial control group risks are drawn from a uniform distribution between 1% and 5% (‘low’ risk), and the lower three plots present the results from the simulated they are drawn from a uniform distribution between 5% and 15% (‘moderately low’ risk). The two left plots present results from scenarios with ‘mild’ heterogeneity (τ2 = 0.05), the middle two results from scenarios with moderate heterogeneity (τ2 = 0.15), and the two right plots results from scenarios with substantial heterogeneity (τ2 = 0.25). (TIFF) [file pone.0025491.s004.tif]

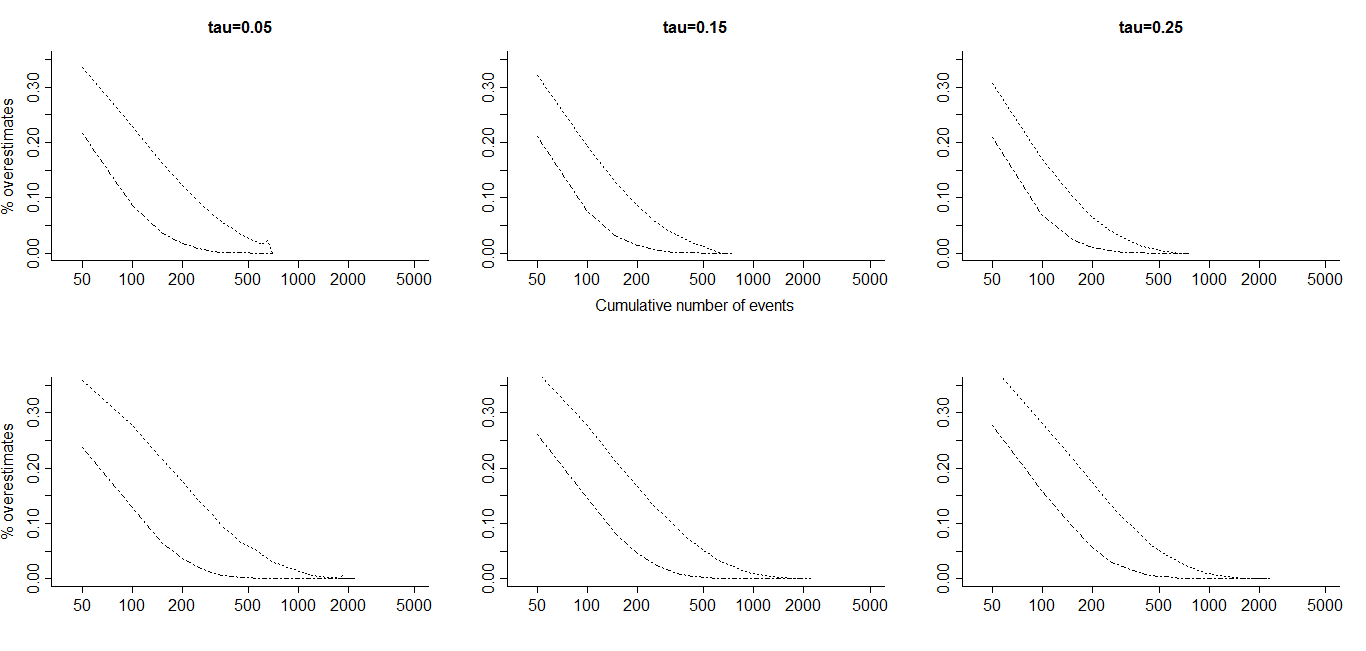

Supplement: Figure S8 — Presents the proportions of pooled intervention effects exceeding a relative risk reduction of 30% (– ▪ – ▪) and 20% (•••••••••) when there is small but potentially important intervention effect (i.e., RRR = 10%), and where the distribution trial sample sizes are our assessment of what constitutes ‘common’ meta-analysis trial size distributions. The proportions are plotted in relation to the cumulative number of events. The upper three plots present the results from the simulated scenarios where the underlying ‘true’ trial control group risks are drawn from a uniform distribution between 1% and 5% (‘low’ risk), and the lower three plots present the results from the simulated they are drawn from a uniform distribution between 5% and 15% (‘moderately low’ risk). The two left plots present results from scenarios with ‘mild’ heterogeneity (τ2 = 0.05), the middle two results from scenarios with moderate heterogeneity (τ2 = 0.15), and the two right plots results from scenarios with substantial heterogeneity (τ2 = 0.25). (TIFF) [file pone.0025491.s008.tif]

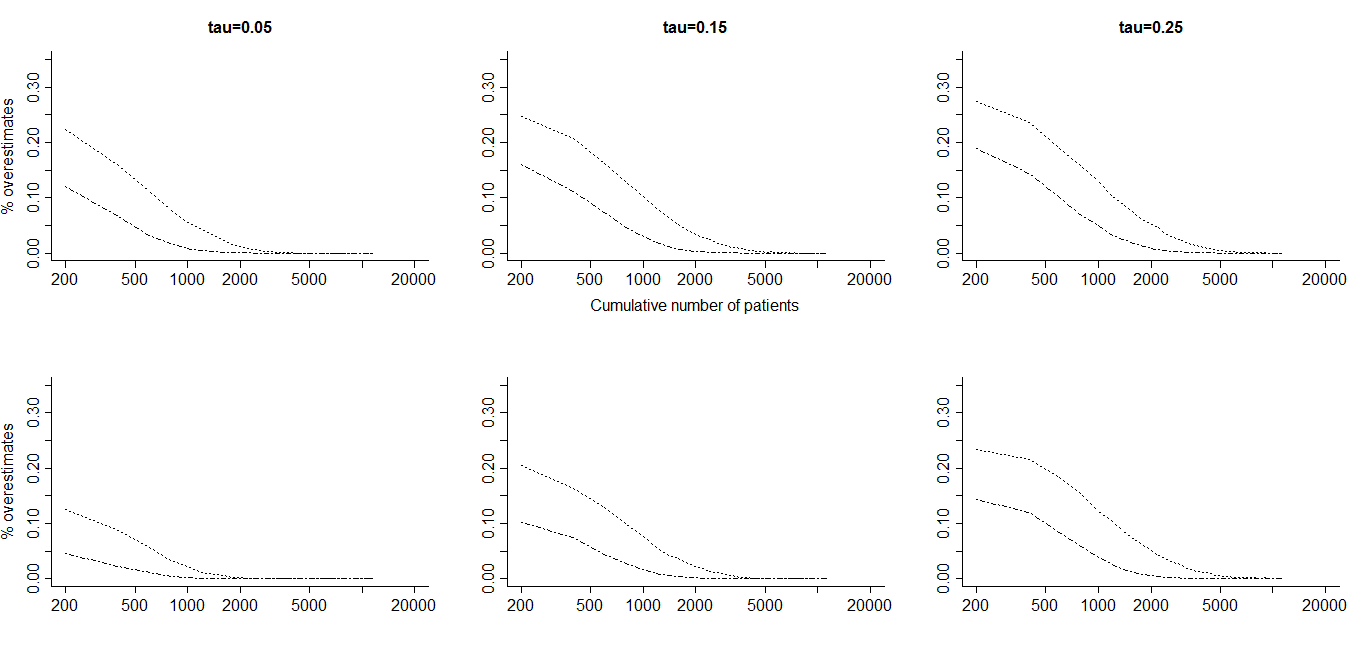

Supplement: Figure S9 — Presents the proportions of pooled intervention effects exceeding a relative risk reduction of 30% (– ▪ – ▪) and 20% (•••••••••) when there is no underlying intervention effect (i.e., RRR = 0%), and where the distribution trial sample sizes are our assessment of what constitutes ‘common’ meta-analysis trial size distributions. The proportions are plotted in relation to the cumulative number of patients. The upper three plots present the results from the simulated scenarios where the underlying ‘true’ trial control group risks are drawn from a uniform distribution between 15% and 40% (‘moderate’ risk), and the lower three plots present the results from the simulated they are drawn from a uniform distribution between 40% and 80% (‘high’ risk). The two left plots present results from scenarios with ‘mild’ heterogeneity (τ2 = 0.05), the middle two results from scenarios with moderate heterogeneity (τ2 = 0.15), and the two right plots results from scenarios with substantial heterogeneity (τ2 = 0.25). (TIFF) [file pone.0025491.s009.tif]

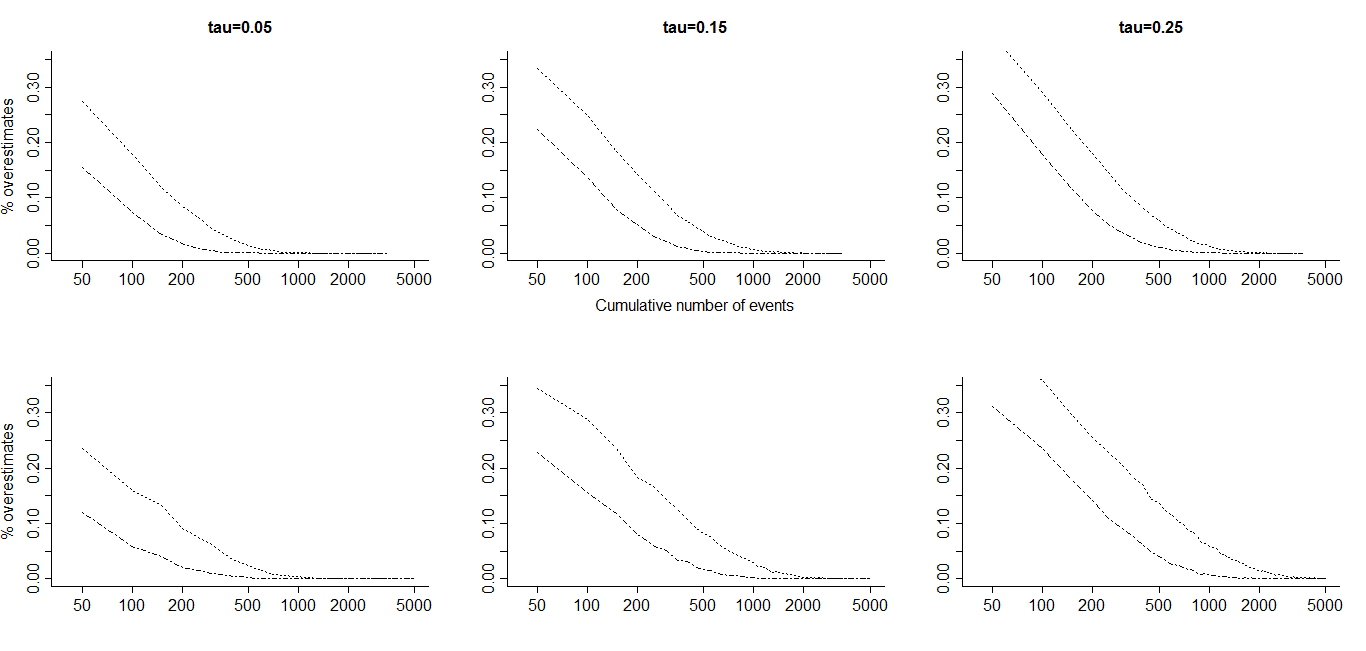

Supplement: Figure S10 — Presents the proportions of pooled intervention effects exceeding a relative risk reduction of 30% (– ▪ – ▪) and 20% (•••••••••) when there is no underlying intervention effect (i.e., RRR = 0%), and where the distribution trial sample sizes are our assessment of what constitutes ‘common’ meta-analysis trial size distributions. The proportions are plotted in relation to the cumulative number of events. The upper three plots present the results from the simulated scenarios where the underlying ‘true’ trial control group risks are drawn from a uniform distribution between 15% and 40% (‘moderate’ risk), and the lower three plots present the results from the simulated they are drawn from a uniform distribution between 40% and 80% (‘high’ risk). The two left plots present results from scenarios with ‘mild’ heterogeneity (τ2 = 0.05), the middle two results from scenarios with moderate heterogeneity (τ2 = 0.15), and the two right plots results from scenarios with substantial heterogeneity (τ2 = 0.25). (TIFF) [file pone.0025491.s010.tif]

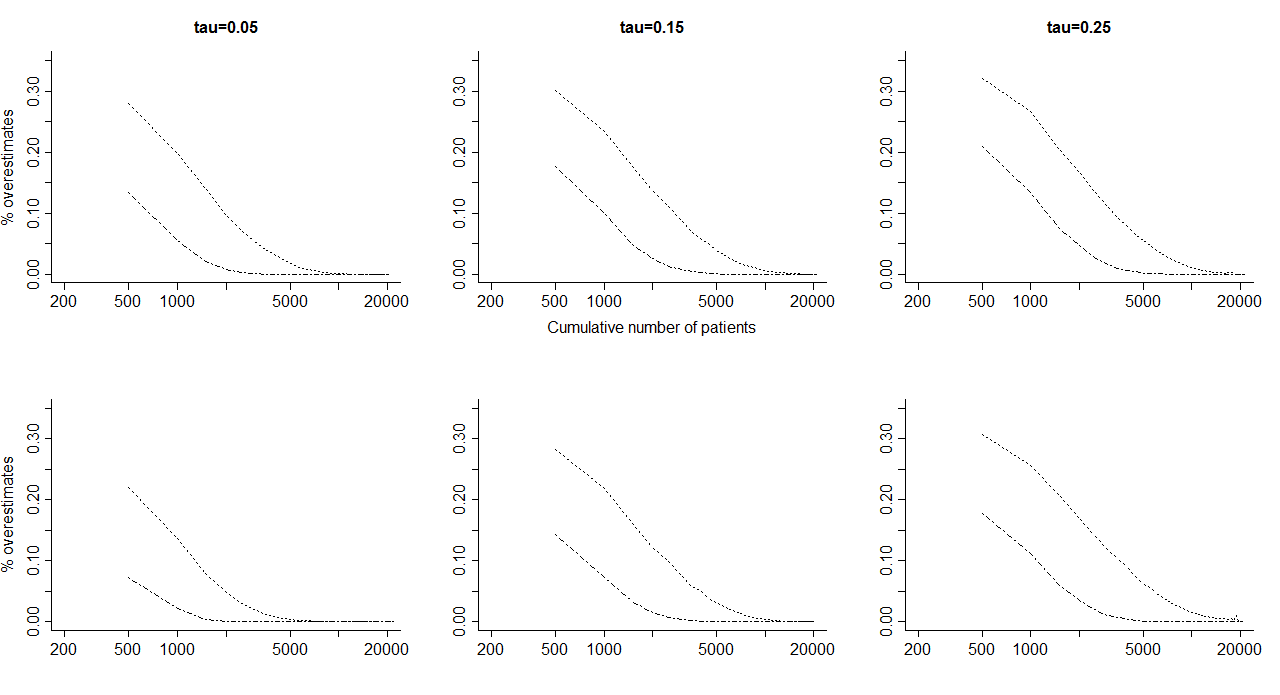

Supplement: Figure S11 — Presents the proportions of pooled intervention effects exceeding a relative risk reduction of 30% (– ▪ – ▪) and 20% (•••••••••) when there is small but potentially important intervention effect (i.e., RRR = 10%), and where the distribution trial sample sizes are our assessment of what constitutes ‘common’ meta-analysis trial size distributions. The proportions are plotted in relation to the cumulative number of patients. The upper three plots present the results from the simulated scenarios where the underlying ‘true’ trial control group risks are drawn from a uniform distribution between 15% and 40% (‘moderate’ risk), and the lower three plots present the results from the simulated they are drawn from a uniform distribution between 40% and 80% (‘high’ risk). The two left plots present results from scenarios with ‘mild’ heterogeneity (τ2 = 0.05), the middle two results from scenarios with moderate heterogeneity (τ2 = 0.15), and the two right plots results from scenarios with substantial heterogeneity (τ2 = 0.25). (TIFF) [file pone.0025491.s011.tif]

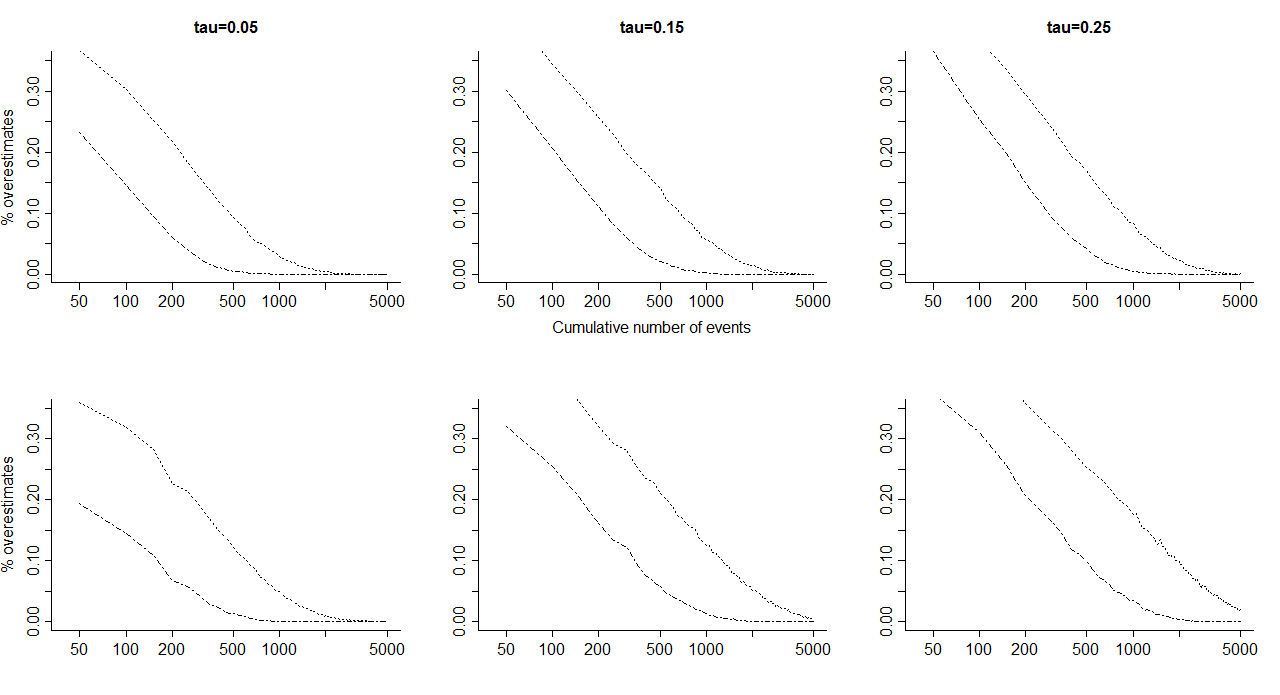

Supplement: Figure S12 — Presents the proportions of pooled intervention effects exceeding a relative risk reduction of 30% (– ▪ – ▪) and 20% (•••••••••) when there is small but potentially important intervention effect (i.e., RRR = 10%), and where the distribution trial sample sizes are our assessment of what constitutes ‘common’ meta-analysis trial size distributions. The proportions are plotted in relation to the cumulative number of events. The upper three plots present the results from the simulated scenarios where the underlying ‘true’ trial control group risks are drawn from a uniform distribution between 15% and 40% (‘moderate’ risk), and the lower three plots present the results from the simulated they are drawn from a uniform distribution between 40% and 80% (‘high’ risk). The two left plots present results from scenarios with ‘mild’ heterogeneity (τ2 = 0.05), the middle two results from scenarios with moderate heterogeneity (τ2 = 0.15), and the two right plots results from scenarios with substantial heterogeneity (τ2 = 0.25). (TIFF) [file pone.0025491.s012.tif]

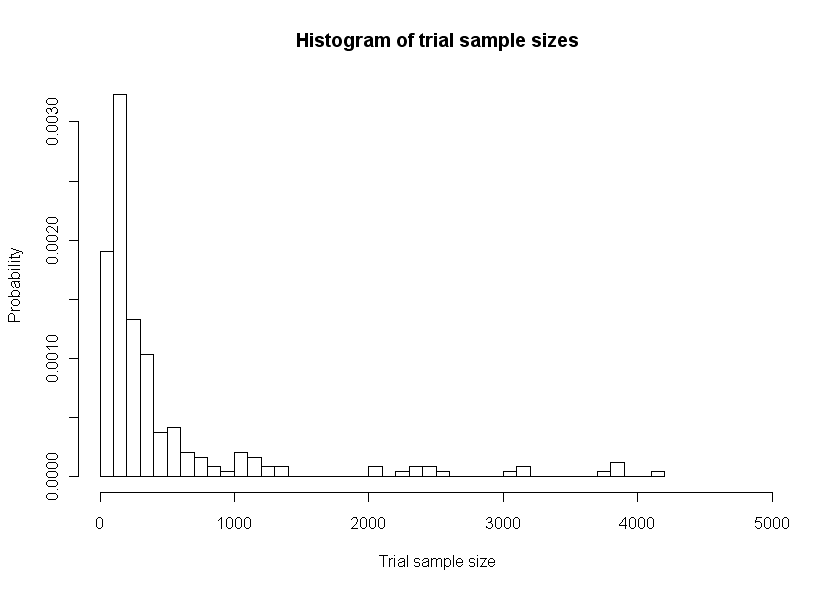

Supplement: Figure S13 — Histogram of trial sample sizes in the surveyed Cochrane heart group meta-analyses. (TIF) [file pone.0025491.s013.tif]
