# Supplementary material for: The Number of Patients and Events Required to Limit the Risk of Overestimation of Intervention Effects in Meta-Analysis—A Simulation Study
Source: PLoS One. 2011 Oct 18;6(10):e25491. doi: 10.1371/journal.pone.0025491 (PMC3196500; doi:10.1371/journal.pone.0025491)
Supplement: Table S1 — Presents the recorded meta-analysis and trial characteristics from the survey of Cochrane Heart Group mortality meta-analyses. The column labeled ‘Quartile’ contains the 25th to 75th percentile interval. The columns labeled ‘Spectrum’ contains the minimum and maximum value observed. The last column contains the DerSimonian-Laird estimate of the between-trial variance (on the log relative risk scale). (DOC) [file pone.0025491.s014.doc]

| **MA** | **Term** | **MA sample size** | | **Trial Sample Size (#patients)** | | | **Trial control group event rate** | | | **Pooled RR (95%CI)** | **τ^2^** |
| --- | --- | --- | --- | --- | --- | --- | --- | --- | --- | --- | --- |
|  |  | #patients | #trials | Median | Quartile | Spectrum | Median | Quartiles | MinMax |  |  |
| 1 | Short | 2799 | 13 | 154 | 101-199 | 47-742 | 0.09 | 0.07-0.12 | 0.02-0.16 | 0.85(0.67-1.07) | 0.00 |
| 2 | Short | 7759 | 8 | 145 | 103-201 | 63-6800 | 0.05 | 0.04-0.06 | 0.01-0.35 | 0.99(0.93-1.06) | 0.00 |
| 3 | Short | 4832 | 15 | 238 | 163-337 | 85-1058 | 0.02 | 0.01-0.04 | 0.005-0.09 | 1.04(0.71-1.53) | 0.00 |
| 4 | Short | 3745 | 6 | 525 | 252-1022 | 62-1285 | 0.02 | 0.01-0.03 | 0.01-0.09 | 0.90(0.64-1.28) | 0.00 |
| 5 | Short | 2312 | 8 | 277 | 215-306 | 88-651 | 0.09 | 0.06-0.11 | 0.01-0.21 | 0.72(0.55-0.95) | 0.00 |
| 6 | Short | 2588 | 12 | 201 | 80-299 | 52-651 | 0.07 | 0.04-0.10 | 0.01-0.22 | 0.76(0.59-0.99) | 0.00 |
| 7 | Short | 2658 | 4 | 139 | 87-716 | 50-2330 | 0.40 | 0.35-0.43 | 0.17-0.48 | 0.84(0.64-1.09) | 0.03 |
| 9 | Short | 8412 | 21 | 164 | 102-311 | 26-3833 | 0.06 | 0.05-0.11 | 0.01-0.24 | 1.17(0.96-1.43) | 0.04 |
| 10 | Short | 6780 | 9 | 298 | 198-477 | 102-3833 | 0.09 | 0.06-0.18 | 0.01-0.22 | 1.18(0.90-1.55) | 0.07 |
| 11 | Short | 7473 | 14 | 187 | 150-329 | 26-3833 | 0.03 | 0.01-0.07 | 0.01-0.11 | 1.30(0.95-1.79) | 0.07 |
|  | | | | | | | | | | | |
| 12 | Long | 2428 | 11 | 165 | 135-275 | 78-503 | 0.09 | 0.06-0.13 | 0.02-0.18 | 0.60(0.40-0.91) | 0.16 |
| 13 | Long | 1799 | 10 | 185 | 115-200 | 34-358 | 0.20 | 0.13-0.28 | 0.06-0.31 | 0.90(0.74-1.10) | 0.00 |
| 14 | Long | 5183 | 4 | 1209 | 355-2150 | 198-2268 | 0.11 | 0.06-0.18 | 0.06-0.20 | 0.98(0.85-1.13) | 0.00 |
| 15 | Long | 18679 | 6 | 3420 | 1217-5018 | 283-5522 | 0.07 | 0.05-0.09 | 0.04-0.17 | 1.00(0.92-1.09) | 0.00 |
| 17 | Long | 33201 | 15 | 360 | 211-1322 | 82-13406 | 0.04 | 0.02-0.11 | 0.01-0.22 | 0.87(0.73-1.03) | 0.03 |
| 18 | Long | 3604 | 6 | 219 | 156-728 | 88-2082 | 0.04 | 0.03-0.08 | 0.02-0.18 | 1.04(0.7-1.52) | 0.04 |
| 19 | Long | 10379 | 22 | 167 | 100-426 | 32-2481 | 0.07 | 0.04-0.14 | 0.01-0.34 | 0.77(0.61-0.99) | 0.12 |
| 20 | Long | 7546 | 11 | 441 | 209-816 | 98-2481 | 0.09 | 0.05-0.10 | 0.01-0.13 | 0.88(0.74-1-04) | 0.00 |
| 21 | Long | 12603 | 20 | 342 | 154-663 | 77-4165 | 0.34 | 0.22-0-45 | 0.08-0.82 | 0.64(0.58-0.71) | 0.02 |
| 22 | Long | 969 | 6 | 185 | 120-191 | 32-275 | 0.09 | 0.05-0.11 | 0.03-0.22 | 1.13(0.70-1.84) | 0.12 |
