# Supplementary material for: The Number of Patients and Events Required to Limit the Risk of Overestimation of Intervention Effects in Meta-Analysis—A Simulation Study
Source: PLoS One. 2011 Oct 18;6(10):e25491. doi: 10.1371/journal.pone.0025491 (PMC3196500; doi:10.1371/journal.pone.0025491)
Supplement: Table S2 — Estimated proportions of trial sample sizes based on the survey of Cochrane Heart Group meta-analysis as well as proportions used in our simulations. (DOC) [file pone.0025491.s015.doc]

| Trial sample size interval | Estimated proportion  for all trials | Estimated proportions excluding trials larger than 5000 patients | Proportions used for simulations |
| --- | --- | --- | --- |
| 20 to 200 | 50.8% | 52.3% | 50.0% |
| 201 to 500 | 26.6% | 27.4% | 27.5% |
| 501 to 1000 | 8.2% | 8.4% | 10.0% |
| 1001 to 2000 | 5.1% | 5.1% | 7.5% |
| 2001 to 5000 | 6.6% | 6.8% | 5.0% |
| 5000 to 10000 | 1.6% | - | - |
| 10000 to 50000 | 1.1% | - | - |
