# Supplementary material for: The Number of Patients and Events Required to Limit the Risk of Overestimation of Intervention Effects in Meta-Analysis—A Simulation Study
Source: PLoS One. 2011 Oct 18;6(10):e25491. doi: 10.1371/journal.pone.0025491 (PMC3196500; doi:10.1371/journal.pone.0025491)
Supplement: Table S3 — Presents the required number of patients and events for the probability of overestimation to drop below 10%, 5% and 1%, in the simulation based on the sensitivity trial size distribution. (DOC) [file pone.0025491.s016.doc]

| **Scenario parameters** | | | |  | **Number of patients required for the probability of overestimation to drop below** | | |  | **Number of events required for the probability of overestimation to drop below** | | |
| --- | --- | --- | --- | --- | --- | --- | --- | --- | --- | --- | --- |
| ***True effect*** | ***Overestimation*** | ***PC*** | ***τ^2^*** |  | **10%** | **5%** | **1%** |  | **10%** | **5%** | **1%** |
| *RRR=0%* | *RRR>30%* | 15%-40% | 0.05 |  | 300 | 500 | 1000 |  | 100 | 150 | 250 |
|  |  |  | 0.15 |  | 400 | 800 | 1500 |  | 150 | 250 | 400 |
|  |  |  | 0.25 |  | 600 | 1000 | 2100 |  | 200 | 300 | 550 |
|  |  | 40%-80% | 0.05 |  | <100 | 200 | 600 |  | 100 | 150 | 350 |
|  |  |  | 0.15 |  | 100 | 600 | 1200 |  | 200 | 350 | 650 |
|  |  |  | 0.25 |  | 500 | 900 | 1800 |  | 300 | 450 | 1000 |
|  | *RRR>20%* | 15%-40% | 0.05 |  | 700 | 1000 | 2100 |  | 200 | 300 | 550 |
|  |  |  | 0.15 |  | 1000 | 1600 | 3300 |  | 300 | 450 | 900 |
|  |  |  | 0.25 |  | 1300 | 2100 | 4200 |  | 400 | 600 | 1100 |
|  |  | 40%-80% | 0.05 |  | 400 | 700 | 1400 |  | 200 | 350 | 750 |
|  |  |  | 0.15 |  | 800 | 1300 | 2600 |  | 450 | 700 | 1450 |
|  |  |  | 0.25 |  | 1200 | 2100 | 4300 |  | 700 | 1100 | 2400 |
|  |  |  |  |  |  |  |  |  |  |  |  |
| *RRR=10%* | *RRR>30%* | 15%-40% | 0.05 |  | 500 | 900 | 1700 |  | 150 | 250 | 450 |
|  |  |  | 0.15 |  | 800 | 1400 | 2600 |  | 250 | 350 | 650 |
|  |  |  | 0.25 |  | 1100 | 1800 | 3400 |  | 300 | 450 | 900 |
|  |  | 40%-80% | 0.05 |  | 300 | 500 | 1100 |  | 200 | 300 | 600 |
|  |  |  | 0.15 |  | 700 | 1100 | 2200 |  | 350 | 600 | 1200 |
|  |  |  | 0.25 |  | 1000 | 1500 | 3200 |  | 500 | 800 | 1600 |
|  | *RRR>20%* | 15%-40% | 0.05 |  | 1900 | 3100 | 6200 |  | 500 | 800 | 1500 |
|  |  |  | 0.15 |  | 2500 | 4100 | 9800 |  | 700 | 1100 | 2150 |
|  |  |  | 0.25 |  | 3500 | 5400 | 11500 |  | 850 | 1350 | 2300 |
|  |  | 40%-80% | 0.05 |  | 1100 | 1900 | 3900 |  | 650 | 1050 | 2000 |
|  |  |  | 0.15 |  | 2300 | 4000 | 9700 |  | 1300 | 2200 | 4300 |
|  |  |  | 0.25 |  | 3400 | 5800 | 11000 |  | 1950 | 3500 | >5000 |
