# Supplementary material for: The Number of Patients and Events Required to Limit the Risk of Overestimation of Intervention Effects in Meta-Analysis—A Simulation Study
Source: PLoS One. 2011 Oct 18;6(10):e25491. doi: 10.1371/journal.pone.0025491 (PMC3196500; doi:10.1371/journal.pone.0025491)
Supplement: Table S4 — Presents the required number of patients and events for the probability of overestimation to drop below 10%, 5% and 1%, in the simulation based on the sensitivity trial size distribution. (DOC) [file pone.0025491.s017.doc]

| **Scenario parameters** | | | |  | **Number of patients required for the probability of overestimation to drop below** | | |  | **Number of events required for the probability of overestimation to drop below** | | |
| --- | --- | --- | --- | --- | --- | --- | --- | --- | --- | --- | --- |
| ***True effect*** | ***Overestimation*** | ***PC*** | ***τ^2^*** |  | **10%** | **5%** | **1%** |  | **10%** | **5%** | **1%** |
| *RRR=0%* | *RRR>30%* | 1%-5% | 0.05 |  | 1500 | 2500 | 4500 |  | 100 | 100 | 200 |
|  |  |  | 0.15 |  | 1500 | 2500 | 4500 |  | 100 | 100 | 200 |
|  |  |  | 0.25 |  | 1500 | 2500 | 4500 |  | 100 | 100 | 200 |
|  |  | 5%-15% | 0.05 |  | 1000 | 1500 | 2500 |  | 100 | 150 | 200 |
|  |  |  | 0.15 |  | 1000 | 1500 | 2500 |  | 100 | 150 | 250 |
|  |  |  | 0.25 |  | 1000 | 1500 | 3000 |  | 100 | 150 | 300 |
|  | *RRR>20%* | 1%-5% | 0.05 |  | 3500 | 5000 | 10500 |  | 150 | 200 | 300 |
|  |  |  | 0.15 |  | 3000 | 4500 | 9000 |  | 150 | 200 | 300 |
|  |  |  | 0.25 |  | 2500 | 4000 | 7500 |  | 100 | 150 | 300 |
|  |  | 5%-15% | 0.05 |  | 1500 | 2500 | 4500 |  | 150 | 250 | 450 |
|  |  |  | 0.15 |  | 2000 | 2500 | 5000 |  | 200 | 250 | 500 |
|  |  |  | 0.25 |  | 2000 | 3000 | 5500 |  | 200 | 300 | 550 |
|  |  |  |  |  |  |  |  |  |  |  |  |
| *RRR=10%* | *RRR>30%* | 1%-5% | 0.05 |  | 2500 | 4000 | 7500 |  | 100 | 150 | 250 |
|  |  |  | 0.15 |  | 2500 | 3500 | 6500 |  | 100 | 150 | 250 |
|  |  |  | 0.25 |  | 2500 | 3500 | 6000 |  | 100 | 150 | 200 |
|  |  | 5%-15% | 0.05 |  | 1500 | 2000 | 3500 |  | 150 | 200 | 350 |
|  |  |  | 0.15 |  | 1500 | 2500 | 4000 |  | 150 | 200 | 400 |
|  |  |  | 0.25 |  | 1500 | 2500 | 4500 |  | 150 | 250 | 450 |
|  | *RRR>20%* | 1%-5% | 0.05 |  | 7500 | 11500 | 19000 |  | 250 | 400 | 650 |
|  |  |  | 0.15 |  | 5000 | 8500 | 17000 |  | 200 | 300 | 450 |
|  |  |  | 0.25 |  | 4500 | 7000 | 14000 |  | 200 | 250 | 350 |
|  |  | 5%-15% | 0.05 |  | 3500 | 5500 | 11500 |  | 350 | 600 | 1150 |
|  |  |  | 0.15 |  | 3500 | 5500 | 12000 |  | 350 | 550 | 900 |
|  |  |  | 0.25 |  | 3500 | 5000 | 11000 |  | 350 | 550 | 900 |
