# Supplementary material for: The Number of Patients and Events Required to Limit the Risk of Overestimation of Intervention Effects in Meta-Analysis—A Simulation Study
Source: PLoS One. 2011 Oct 18;6(10):e25491. doi: 10.1371/journal.pone.0025491 (PMC3196500; doi:10.1371/journal.pone.0025491)
Supplement: Appendix S1 — Presents the conventional random-effects model meta-analysis setup and the DerSimonian-Laird random-effects model. (DOC) [file pone.0025491.s018.doc]

# SUPPORTING INFORMATION 1

## Random-effects model meta-analysis

In the meta-analytic framework, the random-effects model is defined as follows.[1-3] Assume we have k independent trials. Let Y_i_ be the estimate of the effect from the individual trials. Let μ_i_ be the true intervention effect of the *i*th trial, and let σ_i_^2^ denote the variance of μ_i_. The trial specific intervention effects are assumed to vary across trials, with an underlying true effect, μ, and a between-trial variance τ^2^. The random-effects model is defined hierarchically by

Y_i_ = μ_i_ + ε_i_, ε_i_ ~ N(0**,** σ_i_^2^)

(4)

μ_i_ = μ + E_i_, E_i_ ~ N(0**,** τ^2^)

Collapsing the hierarchy, the observed effect measure, Y_i_, is then assumed to satisfy the distributional relationship Y_i_ ~ N(μ**,** σ_i_^2^ + τ^2^), and the trial weights, w_i_^*^, are set as the inverse of the individual trial variances, w_i_^*^ = (σ_i_^2^ + τ^2^)^-1^. Here we use the asterix to indicate that the weights come from the random-effects model (as opposed to the weights coming from a fixed-effect model w_i_ = σ_i_^-2^). In practice, neither σ_1_^2^, …, σ_k_^2^, nor τ^2^ are known. The within-trial variances are typically often estimated using the sampling variances and the between-trial variance τ^2^ is typically obtained using some estimator (see below).

In the random-effects model meta-analysis the overall intervention effect is obtained as a weighted average of the observed intervention effects in the included trials

μ_w_ = (Σ_i_ w_i_^*^ ⋅ Y_i_ )/(Σ_i_ w_i_^*^)

and the variance is estimated as

Var(μ_w_) = 1/(Σ_i_ w_i_^*^)

### DerSimonian-Laird random-effects meta-analysis

In the conventional random-effects model approach proposed by DerSimonian and Laird (DL), the between-trial variance is estimated using a method of moments based estimator.[2] Cochran’s homogeneity test statistic, Q = Σ w_i_ (Y_i_ - μ_w_)^2^, is used as the basis of the DL estimator, as its 1^st^ moment takes the form E(Q) = (k-1) + τ^2^ (S_1_ – (S_2_ / S_1_)), where S_r_ =Σ w_i_^r^, for r = 1,2. Isolating for τ^2^ then yields the expression for the method of moments estimator of the between-trial variance

τ_DL_^2^ = max(0, (Q - k + 1) / (S_1_ – (S_2_ / S_1_)))

# References

1. Higgins JP, Green S (2009) Cochrane Handbook for systematic reviews of interventions, version 5.0.0. John Wiley & Sons.

2. DerSimonian L, Laird N (1986) Meta-analysis in clinical trials. Contr Clin Trials 7: 177-188.

3. Sidik K, Jonkman J (2005) Simple heterogeneity variance estimation for meta-analysis. Journal of Royal Statistical Society(C) 54: 367-384.
