# Supplementary material for: The Number of Patients and Events Required to Limit the Risk of Overestimation of Intervention Effects in Meta-Analysis—A Simulation Study
Source: PLoS One. 2011 Oct 18;6(10):e25491. doi: 10.1371/journal.pone.0025491 (PMC3196500; doi:10.1371/journal.pone.0025491)
Supplement: Appendix S2 — Presents the complete simulation setup as well as the rationale for the choice of parameter distributions and fixed values. (DOC) [file pone.0025491.s019.doc]

# SUPPORTING INFORMATION S2

# Simulation

We aimed to create ‘realistic’ meta-analysis data sets. We first did so by confining our attention to one area of medicine, cardiology, and surveyed all meta-analyses on mortality from the Cochrane Heart Group (Issue 4, 2009) to inform ‘realistic’ simulation parameter settings. However, subsequently realizing an important lack of generalizability from this approach (as a peer reviewer kindly pointed out), we added a number of simulation parameter values and settings to allow for inferences that apply to a greater spectrum of meta-analysis scenarios.

When surveying the meta-analyses from the Cochrane Heart Group, we selected reviews that meta-analyzed results on mortality, including at least 3 trials and 100 events. We separately surveyed meta-analyses in which the median follow-up across trials was between 1 month and 1 year, and between 1 year and 5 years. We did not include meta-analyses including cluster randomized trials in our survey. For each eligible meta-analysis, we recorded the trial sample sizes, the trial control group risk, the pooled relative risks, the associated 95% confidence intervals, and the estimated DerSimonian-Laird between-trial variance (on a log relative risk scale, see Appendix A1 in supporting information). For each eligible meta-analysis, we also recorded the median, minimum, maximum, and the quartiles for the trial sample sizes and control group risks, and summarized these statistics in a table. With the surveyed information, we plotted all trial sample sizes as a histogram.

Tables S1 and S2 and Figure S13 in the supporting information present the data summaries produced from the survey of the Cochrane Heart Group meta-analyses that were used to inform the settings of our simulations.

*Determining simulation parameters*

We simulated binary data meta-analysis scenarios based on distributional assumptions for the trial specific variables: the underlying true intervention effect, μ, the distribution of trial sample sizes, n_i_, the observed control group risks, P_Ci_, and the level of heterogeneity (between-trial variance), τ^2^. The results from the survey of the Cochrane Heart Group meta-analyses and our subsequent considerations informed the values and distributions of each of the above simulation components.

*Distribution of trial sizes*

Figure S13 and the ‘trial sample size’ columns in Table S1 in the supporting information provide an overview of the distribution of trial sample sizes among the surveyed Cochrane Heart Group mortality meta-analyses. After post hoc inspection of Figure S6 we calculated the proportion of trial sizes between 20, 200, 500, 1000, 2000, 5000, 10,000, and 50,000 participants. The estimated proportions are shown in table S2 in the supporting information. For our simulations, we decided only to simulate trials smaller than 5000 patients. We did this because intervention effects from large trials are assumed to come close to the overall average effect, whereas larger variations between intervention effects are typically observed across smaller trials.[1,2] Thus, the random-effects distributional assumptions, such as large between-trial variance, that allow for very large trials to yield vastly different intervention effect estimates do not seem to be representative of meta-analyses in general.

Because larger trials are still rare in many medical areas, we decided to add a distribution of trial sizes to our simulations that more adequately reflected the spectrum and distribution of trial sample sizes that have been reported in the literature and seemed consistent with our own experience with meta-analysis in systematic reviews.[3,4] We decided on a second trial sample size distribution under which there is an 80% chance that a trial will have a sample size between 20 and 200 patients and a 20% chance that a trial will have a sample size between 200 and 500 patients. We have referred to this as a ‘common’ distribution of trial sample sizes in our paper.

*Distribution of control group risks*

In the survey of the Cochrane Heart Group meta-analyses, the median control group risks were generally lower than 10%. Based on table S1 in the supporting information, we assumed two distributions for the ‘true’ trial control group risk: ‘low’ and ‘moderately low’. For low risks we assumed that the underlying trial control group risk would follow a uniform distribution between 1% and 5% (average of 3%). For moderately low risks, we assumed the underlying trial control group risk would follow a uniform distribution between 5% and 15% (average of 10%).

Subsequent considerations led us to additionally consider higher control group risks to increase generalizability of our results. In particular, we assumed that a ‘moderate’ control group risk could be represented by a uniform distribution between 15% and 40% (average of 27.5%), and that a ‘high’ control group risk could be represented by a uniform distribution between 40% and 80% (average of 60%).

*Between-trial variance*

In the Cochrane Heart Group survey, the between-trial variance (heterogeneity) estimates spanned from 0.00 to 0.16, with the most common values being either truncated at 0.00 or in the interval of 0.03 to 0.07. The DerSimonian-Laird estimator is, however, known to underestimate the between-trial variance.[5,6] For this reason, we picked the three between-trial variance values 0.05, 0.15, and 0.25, which were moderately larger than the DerSimonian-Laird estimates observed in the Cochrane Heart Group meta-analysis sample. We believe that these values cover the spectrum in which most between-trial variance estimates fall, and thus, we did not add additional simulation values to those inferred from the survey.

*Underlying true intervention effect*

We considered two hypothetical situations: one where no underlying intervention effect exists (RRR = 0%), and one where a small but possibly unimportant intervention effect exists (RRR = 10%). Of note, the selected underlying true intervention effects were chosen to fit the objectives of this study, but not the results of the Cochrane Heart Group survey.

*The simulation setup*

First, we drew, with probabilities, the interval from which the trial sample size was to be sampled (Table S2 for the Cochrane Heart Group based trial sample sizes, or as given above for `common` trial sample sizes). We then drew the trial sample size, n, from a uniform distribution on the interval that corresponded to the trial size category. The number of patients recruited to each intervention arm was set equal to n/2 (rounded up if n was an odd number).

We drew the trial specific control group risk, P_Ci_*,* from a uniform distribution on one of the intervals given above (corresponding to the given scenario), and subsequently drew the number of observed events in the control group from a binomial distribution e_iC_ ~ bin(n_i_, P_Ci_).

We drew the underlying true trial intervention effects as log relative risks from a normal distribution, ln(RR_i_) ~ N(μ, τ^2^), where μ is the natural logarithm of the underlying ‘true’ relative risk. Lastly, we drew the observed number of events in the intervention group from a binomial distribution e_iE_ ~ bin(n_i_, P_Ei_ ), where P_Ei_  = P_Ci_ ⋅ RR_i_*.* For each scenario, we simulated 20,000 meta-analysis data sets, each including 100 trials.

**References**

1. Glasziou PP, Sheppard S, Brassey J (2010) Can we really rely on the best trial? A comparison of individual trials and systematic reviews. BMC Med Res Methodol 10.

2. Nuesch E, Trelle S, Reichenbach S, Rutjes AWS, Tschannen B, et al. (2011) Small study effects in meta-analysis of osteoarthritis trials: meta-epidemiological study. BMJ 341: c3515.

3. Chan AW, Altman DG (2005) Epidemiology and reporting of randomized clinical trials published in PubMed journals. Lancet 365: 1159-1162.

4. Gluud C (2006) The culture of designing hepato-biliary randomised clincial trials. Journal of Hepatology 44: 607-615.

5. Brockwell S, Gordon I (2001) A comparison of statitical methods for meta-analysis. Statistics in Medicine 20: 825-840.

6. Sidik K, Jonkman J (2007) A comparison of heterogeneity variance estimators in combining results of studies. Statistics in Medicine 26: 1964-1981.
